# Synthesis and Hydrogen Sulfide Releasing Properties of Diaminodisulfides and Dialkoxydisulfides

**DOI:** 10.1021/acsomega.1c02585

**Published:** 2021-06-28

**Authors:** James P. Grace, Ned B. Bowden

**Affiliations:** Department of Chemistry, University of Iowa, W425 Chemistry Building, Iowa City, Iowa 52242, United States

## Abstract

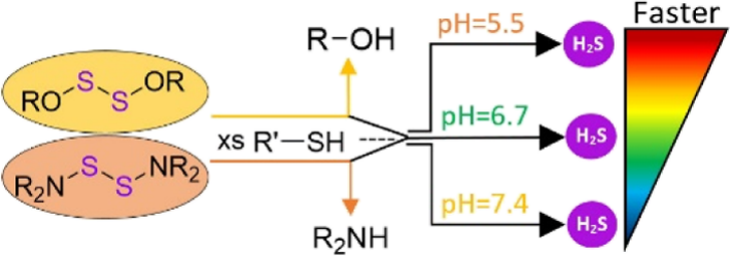

Heterosubstituted disulfides are an understudied class of molecules that have been used in biological studies, but they have not been investigated for their ability to release hydrogen sulfide (H_2_S). The synthesis of two sets of chemicals with the diaminodisulfide (NSSN) and dialkoxydisulfide (OSSO) functional groups was reported. These chemicals were synthesized from commercially available sulfur monochloride or a simple disulfur transfer reagent. Both the diaminodisulfide and dialkoxydisulfide functional groups were found to have rapid rates of H_2_S release in the presence of excess thiol. The release of H_2_S was complete with 10 min, and the only byproducts were conversion of the thiols into disulfides and the amines or alcohols originally used in the synthesis of the diaminodisulfide or dialkoxydisulfide functional groups. These results will allow the design of H_2_S releasing chemicals that also release natural, biocompatible alcohols or amines. Chemicals with the diaminodisulfide and dialkoxydisulfide functional groups may find applications in medicine where a controlled, burst release of H_2_S is needed.

## 1 Introduction

**Figure 1:**
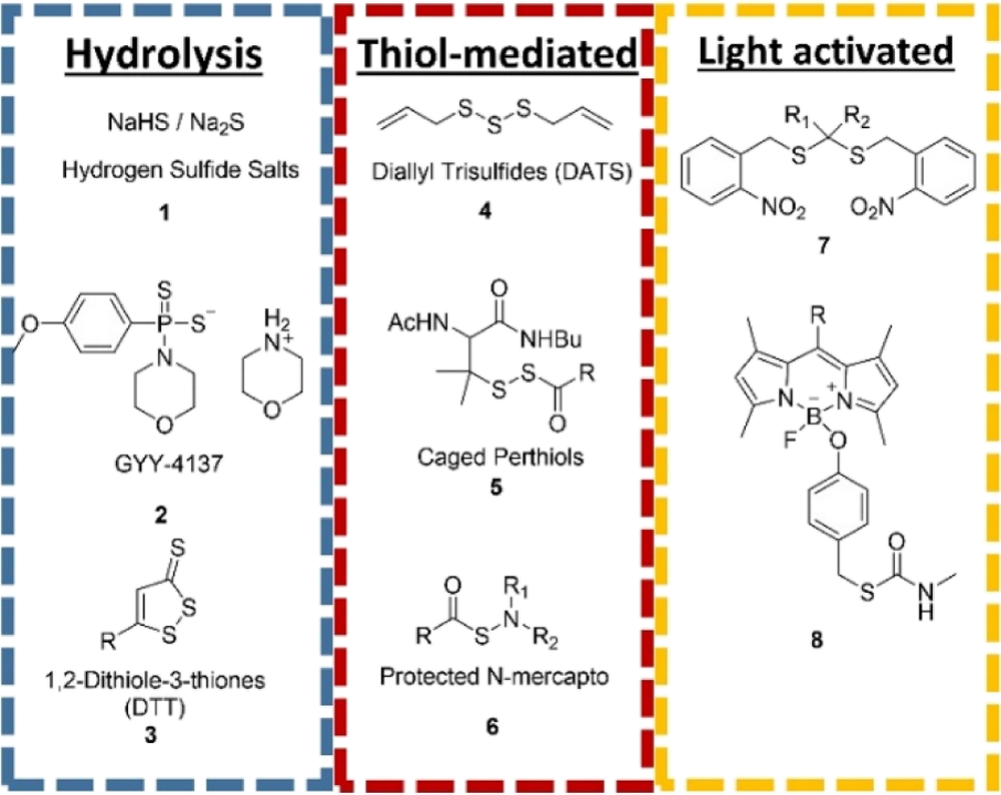
Several common H_2_S donors are shown and classified by their activation mechanisms.

Hydrogen sulfide (H_2_S) is a noxious gas that is commonly associated with potent toxicity at elevated concentrations (>100 ppm) and adverse health effects at single ppm concentrations.^1−7^ However, H_2_S has been found to be endogenously produced in human and plant cells at nanomolar concentrations, and it is involved in modulating numerous intra- and intercellular enzymatic cycles.^3,6,8−12^ H_2_S is widely recognized as the third gasotransmitter, along with nitric oxide and carbon monoxide, that is important to human health.^13−19^ H_2_S is mainly produced by the enzymatic cleavage of disulfides and thiols from cysteine residues by cystathionine-β-synthase and cystathionine-γ-lyase, but it has other endogenous sources as well.^20,21^ Physiological studies of H_2_S include its use as a cardioprotectant against ischemia-reperfusion injury, promoting angiogenesis, modulating hypoxic cell environments, as a chemotherapeutic, and many more.^5,22−28^ There has been a marked increase in publications in the last two decades probing these benefits, yet many studies use the same handful of H_2_S donors. Previous studies used aqueous solutions of inorganic salts NaSH or Na_2_S, but due to the low boiling point of −60 °C for H_2_S, aqueous solutions of H_2_S rapidly release H_2_S into the atmosphere, which led to rapidly changing concentrations of H_2_S and impossible to sustain low therapeutic concentrations.^29^ To address this challenge, chemicals such as GYY-4137 (**2**) or derivatives of 1,2-dithiole-3-thiones (DTT, **3**) that slowly release H_2_S by hydrolysis were developed (Figure 1).^29−32^ GYY-4137 and DTT release H_2_S immediately upon dissolving, but in water, they release H_2_S at concentrations that are 10^5^ times lower than the concentrations of these donors, and the full release of H_2_S may take months.^29,33^ This leads to the use of higher than desired concentrations of these donors in cellular systems.

The importance of H_2_S in many cellular pathways has motivated the synthesis of numerous chemicals that release H_2_S in response to different stimuli. Investigators have developed both nucleophilic and light-activated H_2_S releasing compounds, although the latter is still relatively recent (Figure 1).^4,34^ Most nucleophile-triggered H_2_S donors use thiols such as glutathione (GSH) and cysteine which are found in μM to mM concentrations within the body.^26,34−38^ Modeled after garlic-derived diallyl sulfides [*i.e.*, DATS (**4**)], they produce a perthiol intermediate that releases H_2_S after reaction with biological thiols.^26,37^ In contrast to chemicals such as GYY-4137 that slowly release H_2_S upon hydrolysis, nucleophilic activated chemicals release H_2_S rapidly.^26,34,36−38^ The burst release of H_2_S is advantageous in some biological systems such as for ischemia-reperfusion injury treatment, following stressful cardiac events. In these studies, the rapid release of H_2_S has been shown to modulate the reactive oxygen species generated from reperfusion and minimize damage to the heart.^23,39^ A challenge with the current set of thiol-mediated chemicals that release H_2_S is that their synthesis requires several steps and results in the release of unnatural chemicals. In this paper, we describe the synthesis of new chemicals that address these challenges and further explores the reactions of two functional groups understudied in organic chemistry.

We report the synthesis and H_2_S release of several water-soluble, thiol-mediated, and burst release H_2_S donors based on the functional groups diaminodisulfides and dialkoxydisulfides. These functional groups are poorly studied in organic chemistry, although there are reports of chemicals with these functional groups investigated for their antibacterial properties.^40−42^ Most applications of diaminodisulfides are for use as vulcanizing agents because they break down at elevated temperatures of >150 °C to release radicals.^43^ Heterosubstituted disulfides (XSSX, X = R_2_N and RO) have not been reported to release H_2_S yet stand to join the class of H_2_S donors. Water-soluble versions of these heterosubstituted disulfides were synthesized to investigate their release of H_2_S in aqueous systems with current H_2_S sensing amperometry technology. Diversification of the H_2_S donor library with these compounds opens new applications and improves upon existing applications. The rate of H_2_S release, solubility, conditions needed for H_2_S release, and the byproducts generated are all important considerations when selecting a suitable donor.

## 2 Results and Discussion

### 2.1 Synthesis of Chemicals with OSSO and NSSN Functional Groups

**Figure 2:**
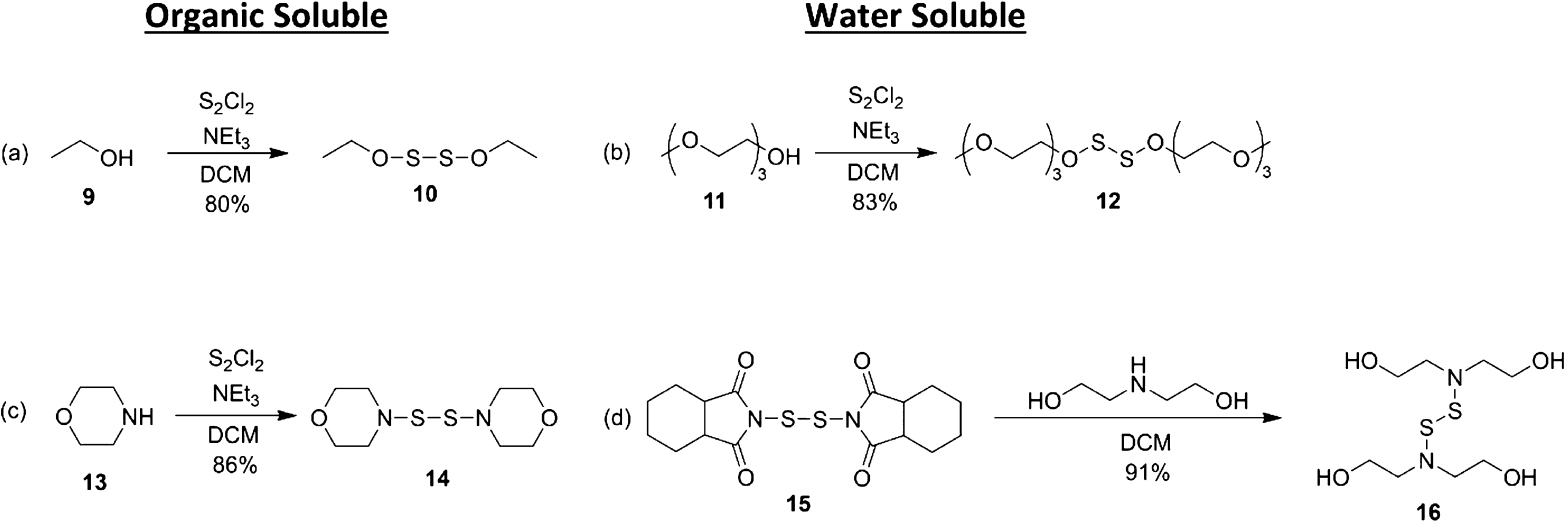
Synthesis of (a) diethoxy disulfide (organic soluble OSSO), (b) triethylene glycol monomethyl ether disulfide (aqueous soluble OSSO), morpholine disulfide (organic soluble OSSO), and diethanolamine disulfide (aqueous soluble NSSN).

Chemicals with the OSSO functional group have been described in the literature.^40,41,44−46^ Their synthesis is typically accomplished by reacting alcohols and sulfur monochloride (S_2_Cl_2_) in the presence of a base. In our work, we synthesized diethoxy disulfide (**10**) and triethylene glycol monomethyl ether disulfide (**12**) in yields of 80% and 83% yield, respectively (Figure 2a,b). The products were purified using column chromatography on silica gel. Although there is no previous report of compound **12** being synthesized in previous literature, the synthetic method employed was known.

Previous reports of the synthesis of chemicals with the NSSN functional group used either S_2_Cl_2_ or disulfide transfer reagents such as **15** that we previously reported.^40,47−51^ Morpholine disulfide (**14**) was synthesized in good yield (86%) by treating morpholine with S_2_Cl_2_ and NEt_3_ at −78 °C (Figure 2c). The reaction of S_2_Cl_2_ with diethanolamine resulted in numerous products due to the reaction of alcohol and amine with S_2_Cl_2_, so a milder disulfide transfer reagent was used. The reaction of diethanolamine with disulfide transfer reagent (**15**) in DCM for 24 h at room temperature resulted in a 91% yield of water-soluble NSSN product **16** (Figure 2d). The byproducts were limited when **15** was used as the S_2_ transfer reagent because it was not sensitive to attack by alcohols, yet it was reactive toward amines.

### 2.2 Stabilities of Chemicals in Organic Solvents

The stabilities of chemicals with the OSSO and NSSN functional groups were investigated. Compound **16** was stable in CD_3_OD and DMSO-*d*_6_ with a variety of different additives including acetic acid, dipropyl amine, hexanol, benzamide, butyronitrile, and *p*-tolyl disulfide (Table S2). After 24 h, >90% of compound **16** was present in each of these experiments as shown by ^1^H NMR spectroscopy. After 24 h in D_2_O, 79% of **16** remained which demonstrated that it slowly decomposed in D_2_O. Compound **10** was also stable in CDCl_3_, and no degradation was observed after 24 h in the presence of acetic acid, hexanol, butyronitrile, benzamide, and *p*-tolyl disulfide (Table S3). Compound **10** slowly reacted with amines, in the presence of butylamine, 82% of **10** remained after 24 h, and in the presence of dipropylamine, 77% remained after 24 h. Importantly, **12** was stable in CD_3_OD over 24 h with no evidence of degradation observed.

### 2.3 Release of H_2_S Triggered by Thiols

**Figure 3:**
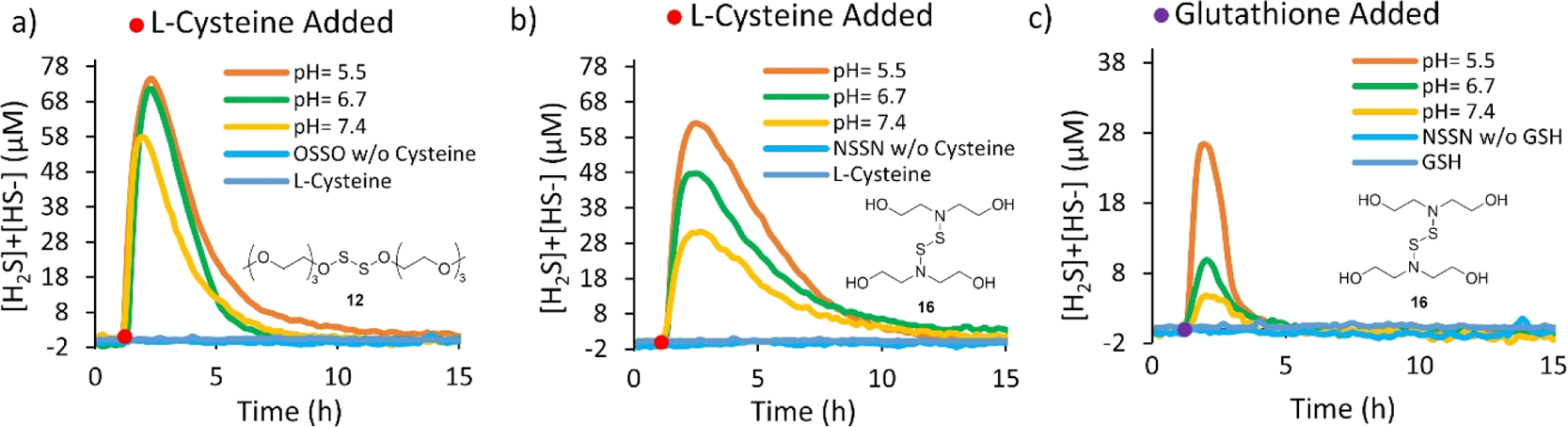
Total sulfide release of (a) **12** (40 μM) and (b) **16** (40 μM) in bis-tris buffer (0.1 M) at pH = 5.5, 6.7, and 7.4. Cysteine (0.6 mM, 15 equiv.) was added after 1 h. (c) Total sulfide release of **16** (40 μM) in bis-tris buffer (0.1 M) at pH = 5.5, 6.7, and 7.4 with the addition of GSH (0.6 mM, 15 equiv) after 1 h.

The thiol-mediated H_2_S release of these compounds was demonstrated by amperometry. The OSSO (**12**) and NSSN (**16**) compounds were studied at a low concentration (40 μM) using a H_2_S microsensor coupled with a pH probe to monitor any fluctuations in the pH of the solution (Figure 3). p*K*_a_ of H_2_S is 7.0, and p*K*_a_ of HS^–^ is reported as >10, so significant amounts of HS^–^ can be present at the pH values used in this work. The total amount of H_2_S and HS^–^ released can be determined by measuring the concentration of H_2_S and pH to allow the concentration of HS^–^ to be calculated. Solutions of selected disulfides were made with bis-tris buffer (0.1 M) at varying values of pH (5.5, 6.7, and 7.4). Bis-tris buffer was selected for its desired pH range from 5.8 to 7.4 allowing us to test all pH values with one buffer to limit variability. Compounds **12** and **16** were also tested for stability and H_2_S release in phosphate-buffered solution (0.1 M, pH = 6.7) (Table S1 and Figure S1, respectively) and found similar H_2_S release profiles compared to the release in bis-tris buffer at the same pH. The chemicals were added to the buffer and stirred for 1 h to ensure that no H_2_S was released, and then, excess l-cysteine (l-Cys) (Figure 3a,b) or GSH (0.6 mM, 15 equiv.) (Figure 3c) was added. The combined release of H_2_S and HS^–^ was monitored in a stirred, uncovered reaction vessel for 18 h. Neither l-Cys, GSH, nor disulfides in the absence of added thiol released any detectable H_2_S. However, both **12** and **16** had a rapid release of H_2_S upon the addition of excess l-Cys or GSH. These measurements were repeated at a range of pH values in order to investigate the effect on H_2_S release. Notably, pH values for all experiments remained within ±0.1 of the starting pH value for the duration of the experiment.

The release of H_2_S was most rapid and resulted in the highest concentration of sulfide [(H_2_S) + (HS^–^)] at the lowest pH investigated for **12** and **16**. For **12**, at a pH of 5.5, the peak H_2_S concentration occured 70 min after the addition of l-Cys, and the peak concentration of sulfide [(H_2_S) + (HS^–^)] was 74 μM. At a pH of 6.7, these values were 80 min and 71 μM, and at a pH of 7.4, these values were 60 min and 58 μM. Similar results were acquired with the NSSN chemical with sulfide peaking times reported after the addition of cysteine (pH = 5.5, 90 min, 62 μM; pH = 6.7, 70 min, 47 μM; and pH = 7.4, 100 min, 31 μM). This data suggests that acidic water promotes a more rapid sulfide release. The release of H_2_S is complex and depends on the protonation of the starting materials and intermediates and the nucleophilicity of thiols and perthiols that may be formed during the reaction. A full understanding of why the pH has a slight effect on the rate of release of H_2_S may be complex. To provide some insights, a proposed mechanism of release of H_2_S will be described in this article. The peaking times of total sulfide concentration are comparable to the time frames of other reported thiol-activated H_2_S donors but release much higher quantities of total sulfide at the same concentration of the starting material.

Interestingly, the peak concentrations of sulfide for both **16** (62 μM) and **12** (74 μM) were greater than that of the initial concentration of disulfides (40 μM). This result demonstrated that the reaction between the NSSN and OSSO chemicals with l-Cys was rapid and that both sulfur atoms in OSSO and NSSN were released as H_2_S or HS^–^, not just one. Importantly, the intracellular concentration of free thiols in the body is frequently reported in the range of 1–10 mM, which is significantly higher than the concentrations tested in this study. Therefore, total sulfide peaking times may shorten with *in vitro*, studies which is a desirable property for a compound designed to release H_2_S quickly.

The release of H_2_S from **16** was investigated with another intracellular thiol, GSH (15 equiv), to demonstrate the effectiveness of a more ubiquitous and commonly tested intracellular thiol (Figure 3c). The reaction was completed in bis-tris buffer (0.1 M) with **16** at a concentration of 40 μM. The sulfide peaking times and concentrations after the addition of GSH were measured at three different pH values (pH = 5.5, 62 min, 26 μM; pH = 6.7, 65 min, 9.7 μM; and pH = 7.4, 67 min, 4.7 μM). Notably, the peak sulfide concentrations are lower with GSH than l-Cys. This is attributed to the lower nucleophilicity of GSH. A quick release of sulfide was still observed, and peaking times were still comparable, but a total sulfide concentration decreased for a more tempered dosing application. The exact intracellular conditions will need to be taken into account when tailoring desired release in future biological studies.

**Figure 4:**
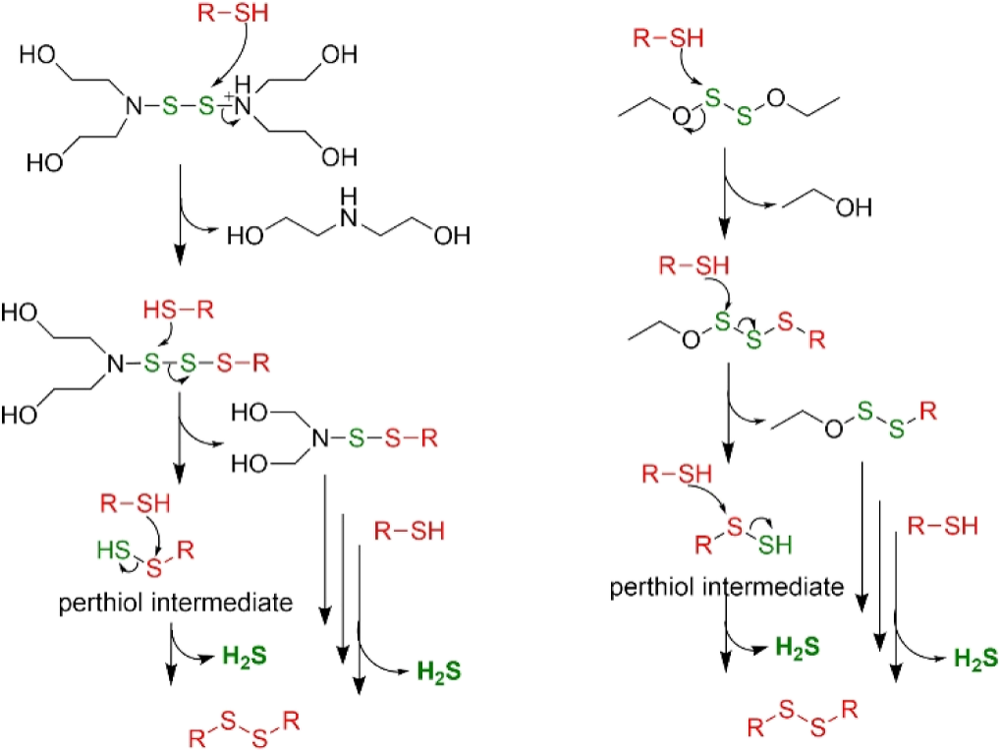
Partial mechanism for the proposed reaction of **16** and **10** with generic thiol (**RSH**) to release H_2_S.

NMR studies were completed to investigate the products of reaction with the OSSO and NSSN chemicals and a thiol (Figure 4). However, l-Cys was not suitable for this study to accurately determine the byproducts by NMR spectroscopy, so 2-mercaptoethanol was chosen as a thiol substitute because of its known ^1^H NMR values of predicted byproducts. Compound **16** was reacted in CH_3_OH with 15 equiv of 2-mercaptoethanol, and the only products observed were diethanolamine and bis(2-hydroxyethyl)disulfide. These products were isolated by column chromatography, and their identities were confirmed by NMR spectroscopy and high-resolution mass spectroscopy (HRMS) analysis. Compound **10** was also reacted with 15 equiv of 2-mercaptoethanol in CD_3_OD, and the reaction was followed by ^1^H NMR spectroscopy. The only products observed were ethanol and bis(2-hydroxyethyl)disulfide. Both of these reactions advanced to high conversions to yield only starting alcohols and amines and disulfide of 2-mercaptoethanol by ^1^H NMR spectroscopy. The reactions of the NSSN and OSSO chemicals with 2-mercaptoethanol were too fast for the kinetics to be measured, but, based on the product distribution, we propose the partial mechanisms, as shown in Figures 4 and S2 and S3. Importantly, these studies confirm that the byproducts of the reactions were parent alcohol or amine and disulfide of the chosen thiol.

### 2.4 Mechanism of the Release of H_2_S

**Figure 5:**
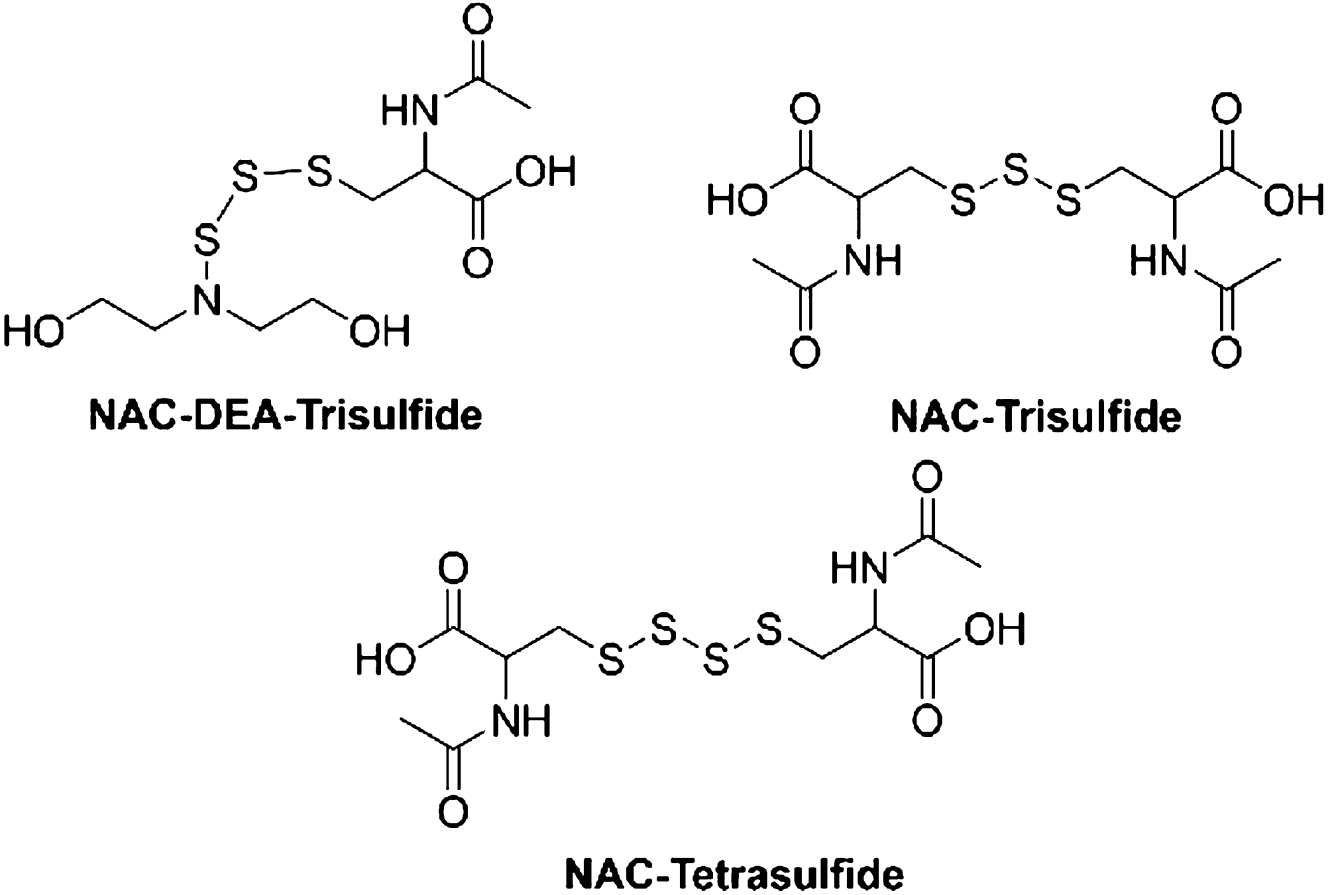
Structures of three of the chemicals found by HRMS in reactions between **16** and NAC.

The HRMS studies were completed to investigate the presence of intermediates of the reaction between **16** and *N*-acetyl cysteine (NAC). These reactions were completed with an excess of NAC (2×, 5×, and 10×) and with *N*-ethyl maleimide (1 equiv) in MeOH to generate and trap intermediates. At time points of 5 min, 20 min, 75 min, 2.5 h, and 24 h, a sample of the reaction was added to a HRMS instrument to investigate which intermediates were present (Figures S4–S20). *N*-Ethyl maleimide was added because it is known to trap perthiols and other reactive thiols, but, unfortunately, no perthiols were trapped with *N*-ethyl maleimide, it was only found to react with NAC. The spectra confirmed the presence of the final products in addition to the presence of trisulfide and tetrasulfide of NAC and NAC–DEA–trisulfide (Figure 5) in all three experiments. The tri- and tetrasulfides of NAC were predicted as possible intermediates (Figures S2 and S3), and these chemicals were expected because H_2_S in the presence of disulfides yields polysulfides through equilibrium reactions.

## 3 Conclusions

In conclusion, heterosubstituted disulfides are a synthetically facile and stable class of thiol-dependent H_2_S releasing compounds that have rapid release profiles measured in minutes compared to the release of H_2_S from chemicals such as GYY-4137 which takes months in water at room temperature to fully degrade.^29,33^ A key advantage of these chemicals is that they release amines and alcohols that were used in their synthesis simplifying the byproduct prediction. By using drugs or biocompatible chemicals with amines or alcohols, the degradation products can be designed to deliver H_2_S and either a pharmaceutical drug or another natural chemical that is known to be safe *in vivo*. A second key advantage of these chemicals is that when synthesized as a prodrug using an amine or alcohol on the drug, these chemicals will release H_2_S and the drug within minutes from each other to ensure that both are released in the same part of the body at the same time. We envision that chemicals with these functional groups can provide controllable, burst release of H_2_S *in vivo* at predictable dosages for therapeutic purposes such as for ischemia-reperfusion injury treatment, following stressful cardiac events where H_2_S must be delivered in minutes to prevent damage to the heart. Additionally, fast-releasing H_2_S donors have the potential as chemotherapeutics due to their cytotoxicity at elevated concentrations. A diversity in H_2_S releasing organic chemicals is essential to explore the pathological and physiological applications for H_2_S, and our compounds possess both a notably fast and complete release of H_2_S along with predictable and safe byproducts.

## 4 Experimental Section

### 4.1 Materials and Supplies

^1^H and ^13^C NMR spectra were recorded on AVANCE 300 MHz and 75 MHz NMR instruments, respectively. Column chromatography was performed using SilicaFlash F60 silica gel (230–400 Mesh). HRMS was conducted on Waters Q-Tof Premier. The H_2_S release was measured using an AMT Analysenmesstechnik amperometric H_2_S microsensor (type III). Phthalimide, *cis*-1,2,3,6-tetrahydrophthalimide, triethylamine, ethanol, diethanolamine, morpholine, and triethylene glycol were purchased from Sigma-Aldrich or Acros Organics and were used as received. Hydrogen gas was purchased from PraxAir. S_2_Cl_2_ was purchased from Aldrich, purified by vacuum distillation over elemental sulfur and charcoal, and stored under N_2_. All solvents were of reagent grade and purchased from Acros Organics or Sigma-Aldrich. All yields reported are isolated yields unless reported otherwise.

### 4.2 Diethoxy disulfide (**10**)

A solution of triethylamine (2.53 g, 25.0 mmol) and ethanol (1.16 g, 25.1 mmol) in CH_2_Cl_2_ (40 mL) was cooled to 0 °C for 20 min. S_2_Cl_2_ (1.69 g, 12.5 mmol) in CH_2_Cl_2_ (10 mL) was added dropwise to the reaction for 16 min and was stirred at 0 °C for 1 h. The reaction was quenched with water, washed with 2 × 50 mL portions of water, and washed once with 50 mL of saturated NaCl. The organic layer was dried over anhydrous magnesium sulfate and evaporated to give an orange oil. The product was purified by column chromatography on silica gel, eluting with 5% ethyl acetate in hexanes to yield a light yellow oil (1.53 g, 80%). ^1^H NMR (CDCl_3_): δ 1.29 (t, 6H), 3.84 (m, 2H), 3.98 (m, 2H). ^13^C NMR (CDCl_3_): δ 15.55, 71.06. HRMS: (M + Na) calcd for C_4_H_10_O_2_S_2_, 154.0122; found, 154.0134.

### 4.3 Triethylene glycol monomethyl ether disulfide (**12**)

A solution of triethylamine (2.53 g, 24.9 mmol) and triethylene glycol monomethyl ether (4.11 g, 25.0 mmol) in CH_2_Cl_2_ (40 mL) was cooled to 0 °C for 20 min. S_2_Cl_2_ (1.69 g, 12.5 mmol) in CH_2_Cl_2_ (10 mL) was added dropwise to the reaction for 10 min and was continuously stirred at 0 °C for 1 h and at room temperature for an additional hour. The reaction was quenched with water and washed with an additional 2 × 50 mL portions of water and then saturated NaCl solution. The organic layer was dried over anhydrous magnesium sulfate and evaporated to give an orange oil. The product was purified by column chromatography on silica gel, eluting with 5% MeOH solution in DCM to yield a dark yellow oil (4.04 g, 83%). ^1^H NMR (CDCl_3_): δ 3.38 (s, 6H), 3.55 (m, 4H), 3.66 (m, 12H), 3.70 (m, 4H), 3.94 (m, 2H), 4.08 (m, 2H). ^13^C NMR (CDCl_3_): δ 59.01, 70.02, 70.58, 70.60, 70.62, 70.72, 71.96, 74.38. HRMS: (M + Na) calcd for C_14_H_30_O_8_S_2_Na, 413.1280; found, 413.1284.

### 4.4 Bis(*cis*-1,2,3,4,5,6-hexahydrophthalimide)disulfide (**15**)

We followed a procedure previously described in the literature.^1^ A solution of triethylamine (5.12 g, 50.5 mmol) and *cis*-1,2,3,4,5,6-hexahydrophthalimide (7.05 g, 46.0 mmol) in CH_2_Cl_2_ (100 mL) was cooled to −90 °C in an acetone/N_2(l)_ bath. S_2_Cl_2_ (3.11 g, 23.0 mmol) in CH_2_Cl_2_ (20 mL) was added dropwise to the reaction for 12 min and was stirred at −90 °C for 30 min. The reaction was quenched with ice cold water and washed with 4 × 50 mL portions of NaOH solution (0.2 M) and saturated NaCl solution. The organic layer was dried over anhydrous magnesium sulfate and evaporated to give a brown solid. The product was purified by recrystallization by dissolving in minimal boiling ethyl acetate and precipitating with boiling hexanes (2.278 g, 27%). ^1^H NMR (CDCl_3_): δ 1.49 (m, 4H), 1.88 (m, 4H), 2.98 (m, 2H). ^13^C NMR (CDCl_3_): δ 21.85, 22.26, 24.12, 40.83, 177.65.

### 4.5 Bis(diethanolamine)disulfide (**16**)

Bis(*cis*-1,2,3,4,5,6-hexahydrophthalimide)disulfide (2.80 g, 7.59 mmol) in CH_2_Cl_2_ (10 mL) was added to a solution of diethanolamine (2.80 g, 7.6 mmol) in CH_2_Cl_2_ (25 mL) at room temperature and stirred for 24 h. A white solid was collected without further purification (0.94 g, 91%). ^1^H NMR (CD_3_OD): δ 2.99 (t, 4H), 3.72 (t, 4H). ^13^C NMR (CD_3_OD): δ 60.56, 61.46. HRMS: (M + Na) calcd for C_4_H_10_N_2_O_4_S_2_Na, 295.0762; found, 295.0758.

### 4.6 Dimorpholine disulfide (**14**)

A solution of triethylamine (2.23 g, 22.1 mmol) and morpholine (1.76 g, 20.2 mmol) in CH_2_Cl_2_ (100 mL) was cooled to −90 °C in an acetone/N_2_ bath. S_2_Cl_2_ (1.35 g, 10.0 mmol) in CH_2_Cl_2_ (5 mL) was added dropwise to the reaction for 8 min and was stirred at −90 °C for 30 min. The reaction was quenched with ice cold water and washed with 2 × 20 mL portions of NaOH solution (0.2 M) and saturated NaCl solution. The organic layer was dried over anhydrous magnesium sulfate and evaporated to give a brown solid. The product was purified by recrystallization by dissolving in minimal boiling ethyl acetate and precipitating with boiling hexanes (2.03 g, 86%). The NMR spectra of this chemical matched that of a previous report.^2^^1^H NMR (CDCl_3_): δ 2.83 (t, 4H), 3.74 (t, 4H). ^13^C NMR (CDCl_3_): δ 55.8, 67.3.

### 4.7 Amperometry Experiments for the Detection of Thiol-Mediated H_2_S Release

Preparation of buffer: a solution of 2-(bis(2-hydroxyethyl)imino)-2(hydroxymethyl-1,3-propanediol) (20.93 g, 0.1 mol) in water (750 mL) was prepared. HCl was added dropwise while stirring until the desired pH was achieved (5.5, 6.7, and 7.4). The buffer was transferred to a 1 L volumetric flask and diluted to 1 L. The buffer was used in H_2_S detection experiments without further modification.

A stock solution of diethanolamine disulfide (27.2 mg, 0.1 mmol) in water (10 mL) was prepared. A portion of the diethanolamine disulfide stock solution (0.30 mL) was diluted in 0.1 M bis-tris buffer (75 mL, 40 μM, pH = 5.5, 6.7, and 7.4) in a 100 mL jar equipped with a stir bar. The concentration of total sulfide (H_2_S + HS^–^) was recorded for 1 h while stirring, and then, a stock solution of l-Cys (0.56 mL of 80 mM stock solution) was added to yield a final concentration of l-Cys of 0.60 mM (15 equiv to diethanolamine disulfide). The concentration of total sulfide (H_2_S + HS^–^) was recorded for an additional 17 h.

A stock solution of triethylene glycol monomethyl ether disulfide (TEG-DS) (39.0 mg, 0.1 mmol) in water (10 mL) was prepared. A portion of the TEG-DS stock solution (0.30 mL) was diluted in 0.1 M bis-tris buffer (75 mL, 40 μM, pH = 5.5, 6.7, and 7.4) in a 100 mL jar equipped with a stir bar. The concentration of total sulfide (H_2_S + HS^–^) was recorded for 1 h while stirring, and then, a stock solution of l-Cys (0.56 mL of 80 mM stock solution) was added to yield a final concentration of l-Cys of 0.60 mM (15 equiv to TEG-DS). The concentration of total sulfide (H_2_S + HS^–^) was recorded for an additional 17 h.

## Supporting Information Available

Detailed experimental procedures, complete ^1^H and ^13^C NMR spectroscopic data of synthesized compounds, description of the HRMS experiments, and HRMS data (PDF)
