# Supplementary material for: Synthesis and Hydrogen Sulfide Releasing Properties of Diaminodisulfides and Dialkoxydisulfides
Source: ACS Omega. 2021 Jun 28;6(27):17741–7. doi: 10.1021/acsomega.1c02585 (PMC8280695; doi:10.1021/acsomega.1c02585)
Supplement: Supplementary file 1 — ao1c02585_si_001.pdf [file ao1c02585_si_001.pdf]

# Supporting Information

Synthesis and Hydrogen Sulfide Releasing Properties of Diaminodisulfides and Dialkoxydisulfides

James P. Grace and Ned B. Bowden*
University of Iowa
Department of Chemistry
W425 Chemistry Building
Iowa City IA, 52242
James-grace@uiowa.edu
ned-bowden@uiowa.edu

**Table of Contents**


| | |
|---|---|
| Synthetic procedures | S3 |
| Amperometric Procedures for $H_2S$ detection | S3 |
| Degradation Studies of both NSSN and OSSO compounds | S3-S4 |
| Stability studies of NSSN and OSSO compounds | S4-S6 |
| Full proposed mechanism of $H_2S$ release | S7 |
| Intermediate Determination by Mass Spectra | S8-S19 |
| Spectroscopic Data | S20-S25 |
| References | S26 |

**cis-1,2,3,4,5,6-Hexahydrophthalimide:**

We followed a procedure previously described in the literature.[1] Pd/C (10% w/w) (0.51 g, cat.) was added to a solution of cis-1,2,3,6-Tetrahydrophthalimide (25.03 g, 165 mmol) in $CH_2Cl_2$ (200 mL) in a Parr reactor equipped with a stir bar. The Parr reactor was assembled and charged with hydrogen gas (1000 psi) and allowed to stir at room temperature for 6 days. The reaction was filtered and the filtrate evaporated to yield a white crystalline solid (23.65 g, 94%). $^1$H NMR ($CDCl_3$): δ 1.47 (m, 4H), 1.80 (m, 2H), 1.86 (m, 2H), 2.92 (m, 2H), 8.51 (broad s, 1H). $^{13}$C NMR ($CDCl_3$): δ 21.89, 23.91, 41.08, 180.56.

**Degradation studies of diethanolamine disulfide (16) with 2-mercapto ethanol:**

Diethanolamine disulfide (0.27 g, 1 mmol) was dissolved in MeOH (20 mL). 2-Mercaptoethanol (1.17 g, 15 mmol) was added in one portion and allowed to stir for 24 h. Three products were observed by $^1$H NMR spectroscopy, and they were isolated by silica gel column chromatography using 5% MeOH in DCM. Diethanolamine and excess 2-mercapto ethanol were identified by known $^1$H NMR and $^{13}$C NMR chemical shift values. The third isolated product bis(2-hydroxyethyl)disulfide was additionally confirmed by HRMS. Individual data for each isolated product given below.

Diethanolamine: (0.20 g, 95% isolated yield) $^1$H NMR: δ 2.75 (t, 2H), 3.67 (t, 2H); $^{13}$C NMR: δ 50.72, 59.92.

Bis(2-hydroxyethyl)disulfide: (0.29 g, 97% isolated yield) $^1$H NMR: δ 2.83 (t, 2H), 3.78 (t, 2H); $^{13}$C NMR: δ 40.89, 59.93. HRMS calc. for $C_2H_6O_2S_2$: 154.0122; Found: 154.0134.

**Degradation studies of diethoxy disulfide (10) and 2-mercapto ethanol:**

Diethoxy disulfide (0.15 g, 1 mmol) was dissolved in MeOH (30 mL). 2-Mercapto ethanol (1.17 g, 15 mmol) was added in one portion with MeOH (20 mL). The reaction was stirred at room temperature. Aliquots were extracted at various time points and solvent removed under reduced pressure. $^1$H NMR spectra were taken at these time points (24 h, 48 h, 72 h). The NMR spectra was compared to the data from the previously isolated 2-mercaptoethoxy disulfide to confirm products. These products were not isolated due to ethanol's volatility. Individual data for each observed product is given below.

2-mercaptoethanol: $^1$H NMR: $^1$H NMR: δ 2.59 (t, 2H), 3.64 (t, 2H); $^{13}$C NMR: δ 27.40, 64.04.

Ethanol: $^1$H NMR: δ 3.63 (q, 4H), 1.19 (t, 6H); $^{13}$C NMR: δ 18.13, 57.79.

bis(2-hydroxyethyl)disulfide: $^1$H NMR: δ 2.83 (t, 2H), 3.78 (t, 2H); $^{13}$C NMR: δ 15.56, 71.07.

**Stability studies of bis(triethylene glycol monomethyl ether) disulfide (12) and diethanolamine disulfide (16) in phosphate buffered solutions (PBS) (pH = 5.5, 6.7, 7.4)**

Compounds **10** or **12** (0.05 mmol) were added to a vial and dissolved in 20 mL of phosphate buffer solution (0.1 M, pH = 5.5, 6.7, or 7.4). At 24 hours an aliquot (0.50 mL) was extracted and water was removed under a stream of $N_2$. 1,3-Dinitrobenzene (0.0167 g, 0.05 mmol) was added to the NMR tube as an internal standard for $^1$H NMR spectroscopy analysis. The percent degradation was determined by tracking the α-hydrogens to the disulfide with the internal standard.

**Table S1**. Stability studies of **16** and **12** at three pH values in phosphate buffered solutions (0.1 M).

| Phosphate Buffer pH | NSSN %NSSN Remaining (24 h) | OSSO %OSSO Remaining (24 h) |
|---|---|---|
| 5.5 | 96 | 90 |
| 6.7 | 69 | 79 |
| 7.4 | 34 | 59 |

**$H_2S$ release studies of bis(triethylene glycol monomethyl ether) disulfide (12) and diethanolamine disulfide (16) in phosphate buffered solution (PBS) (pH = 6.7)**

A stock solution of diethanolamine disulfide (27.2 mg, 0.1 mmol) in water (10 mL) was prepared. A portion of the diethanolamine disulfide stock solution (0.30 mL) was diluted in 0.1 M phosphate buffer (75 mL, 40 µM, pH= 6.7) in a 100 mL jar equipped with a stir bar. The concentration of total sulfide ($H_2S$ + $HS^-$) was recorded for 1h while stirring, and then a stock solution of *L*-cysteine (0.56 mL of an 80 mM stock solution) was added to yield a final concentration of *L*-cysteine of 0.60 mM (15 eq. to diethanolamine disulfide). The concentration of total sulfide ($H_2S$ + $HS^-$) was recorded for an additional 17 h (Figure S1).

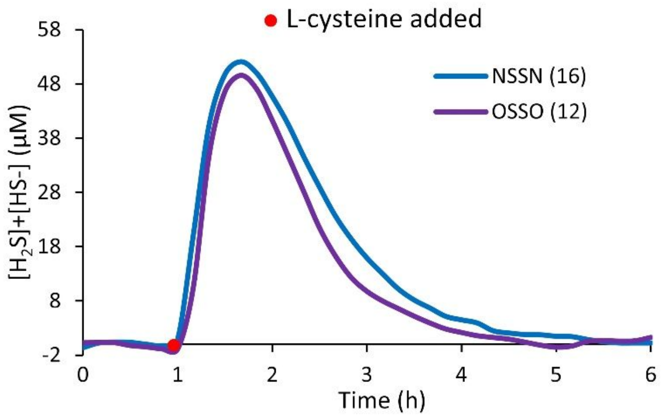


**Figure S1**. The total sulfide release of **16** and **12** (40 µM) in phosphate buffer (0.1 M, pH= 6.7) is shown. Cysteine (6 mM, 15 equiv.) was added after 1 h as indicated by the red dot.

**Stability studies of diethoxy disulfide (10) and bis(triethylene glycol monomethyl ether) disulfide (12) (Table S2):**

Compounds 10 or 12 (0.1 mmol) were added to a vial and dissolved in 0.5 mL $CDCl_3$. 1,3-Dinitrobenzene (0.0167 g, 0.1 mmol) was added to the NMR tube as an internal standard for $^1H$ NMR analysis. If an additive was used, 1 equivalent (0.1 mmol) was added to the experiment immediately before the initial NMR. $^1H$ NMR spectra were taken at 2 h and 24 h to track degradation by comparison of the α-hydrogens to the disulfide with the internal standard.

**Stability studies of diethanolamine disulfide (16) (Table S3):**

Diethanolamine disulfide (0.0272 g, 0.1 mmol) was added to a vial and dissolved in 0.5 mL $d_6$-DMSO. 1,3-Dinitrobenzene (0.0167 g, 0.1 mmol) was added to the NMR tube as an internal standard for NMR analysis. If an additive was used, 1 equivalent (0.1 mmol) was added to the experiment

immediately before the initial NMR spectrum. $^{1}$H NMR spectra were taken at 2 h and 24 h to track degradation by comparison of the α-hydrogens to the disulfide with the internal standard.

**Table S2.** Stability studies of **10** and **12** with exposure to different additives.

| Experiment | OSSO | Additive | Solvent | Remaining OSSO after 24 h (%) |
|---|---|---|---|---|
| **1** | 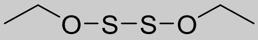 | No Additive | $CDCl_3$ | >97 |
| **3** | 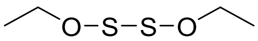 | 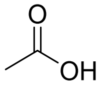 | $CDCl_3$ | >97 |
| **4** | 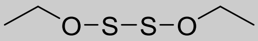 | 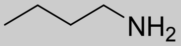 | $CDCl_3$ | 82 |
| **5** | 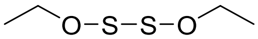 | 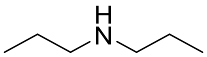 | $CDCl_3$ | 77 |
| **6** | 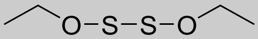 | 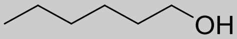 | $CDCl_3$ | >97 |
| **7** | 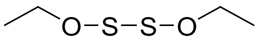 | 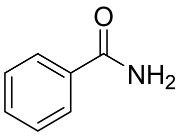 | $CDCl_3$ | >97 |
| **8** | 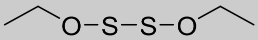 | 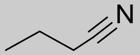 | $CDCl_3$ | >97 |
| **9** | 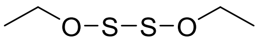 | 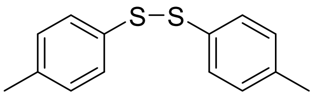 | $CDCl_3$ | >97 |
| **11** | 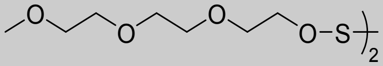 | No Additive | $CD_3OD$ | >97 |

**Table S3.** Stability studies of **16** with exposure to aqueous conditions and various functional group additives.

| **Experiment** | **NSSN** | **Additive** | **Solvent** | **Remaining OSSO after 24 h (%)** |
|---|---|---|---|---|
| **1** | 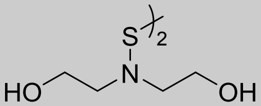 | No Additive | CD$_3$OD | 93 |
| **2** | 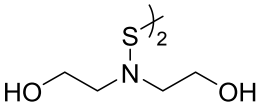 | No Additive | DMSO-d$_6$ | 94 |
| **3** | 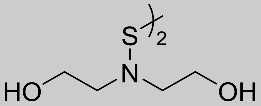 | 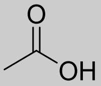 | DMSO-d$_6$ | 92 |
| **5** | 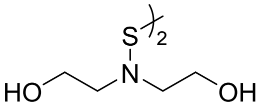 | 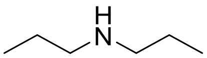 | DMSO-d$_6$ | 81 |
| **6** | 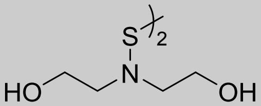 | 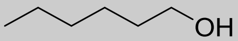 | DMSO-d$_6$ | 91 |
| **7** | 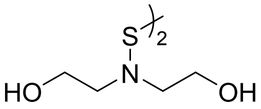 | 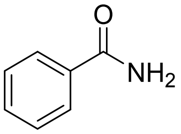 | DMSO-d$_6$ | 94 |
| **8** | 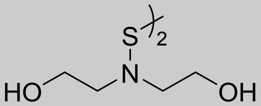 | 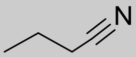 | DMSO-d$_6$ | 91 |
| **9** | 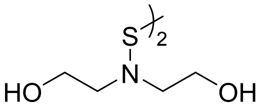 | 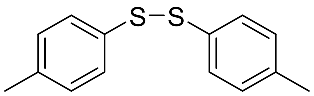 | DMSO-d$_6$ | 92 |
| **10** | 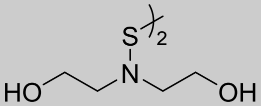 | No Additive | D$_2$O | 79 |
| **11** | 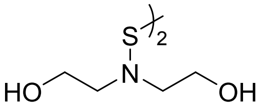 | No Additive | CD$_3$OD | 99 |

**Proposed mechanism of reaction of 12 and 16 with 2-mercaptoethanol as model thiol**

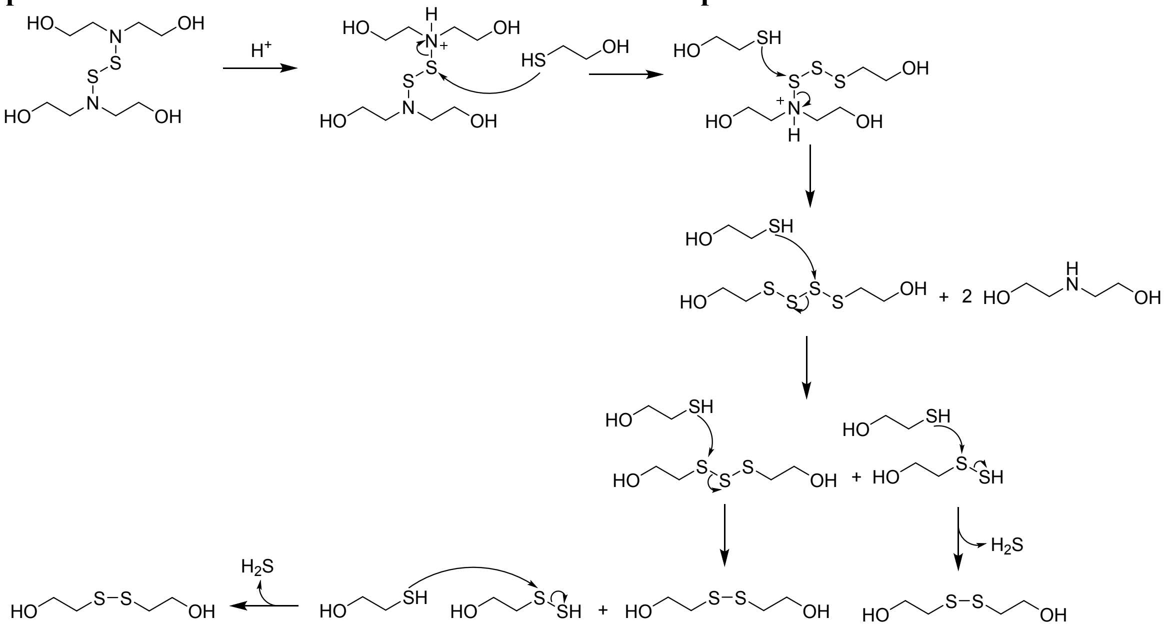

**Figure S2.** The proposed degradation mechanisms in the presence of a thiol (2-mercaptoethanol) of **16** is shown.

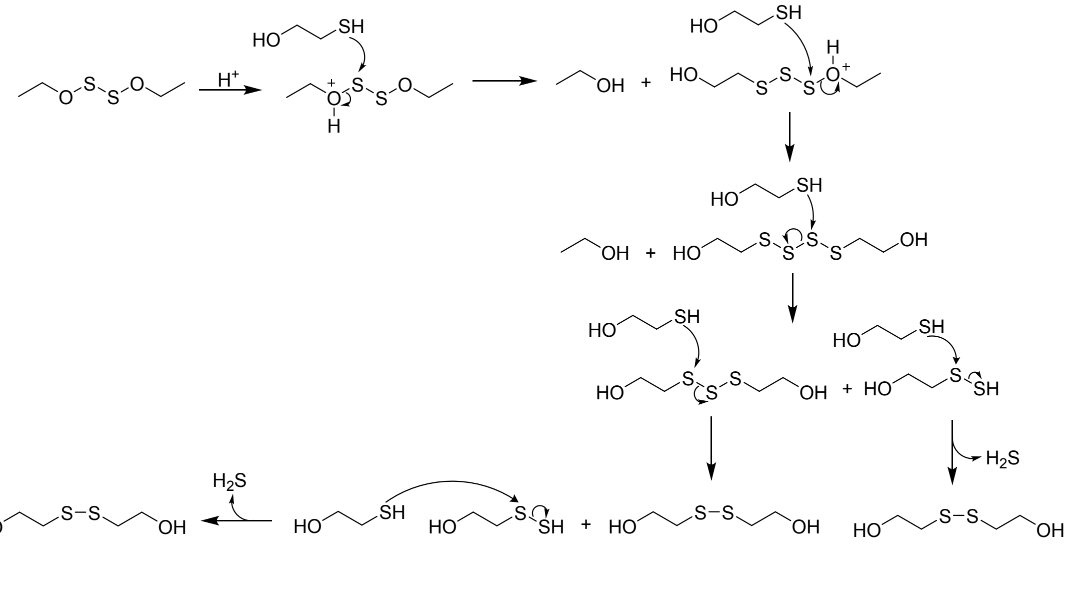

**Figure S3.** The proposed degradation mechanisms in the presence of a thiol (2-mercaptoethanol) of **12** is shown.

**High Resolution Mass Spectroscopy Data**

**General Reaction Scheme (2x, 5x, and 10x equivalents of NAC).** Three stock solutions of **16** (13.5 mg, 0.05 mmol, 0.1 mM) and NEM (6.6 mg, 0.05 mmol, 1 equiv.) in MeOH (5.0 mL) were set up. To these reactions *N*-zcetyl cysteine (NAC) 16.2 mg (0.1 mmol), 40.7 mg (0.25 mmol), or 81.4 mg (0.5 mmol) were added and the reactions were stirred at room temperature. Aliquots (50 µL) were removed from each reaction at designated time points (5 min, 20 min, 75 min, 2.5 h, 24 h) and diluted with additional optical grade MeOH to appropriate concentrations for HRMS. The samples were injected into an ESI-HRMS spectrometer immediately after diluting.

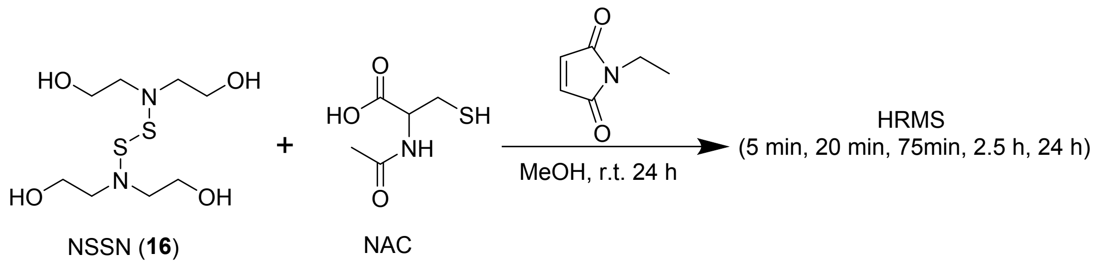


**Figure S4.** General reaction scheme for HRMS studies.

**Observed Products in HRMS**

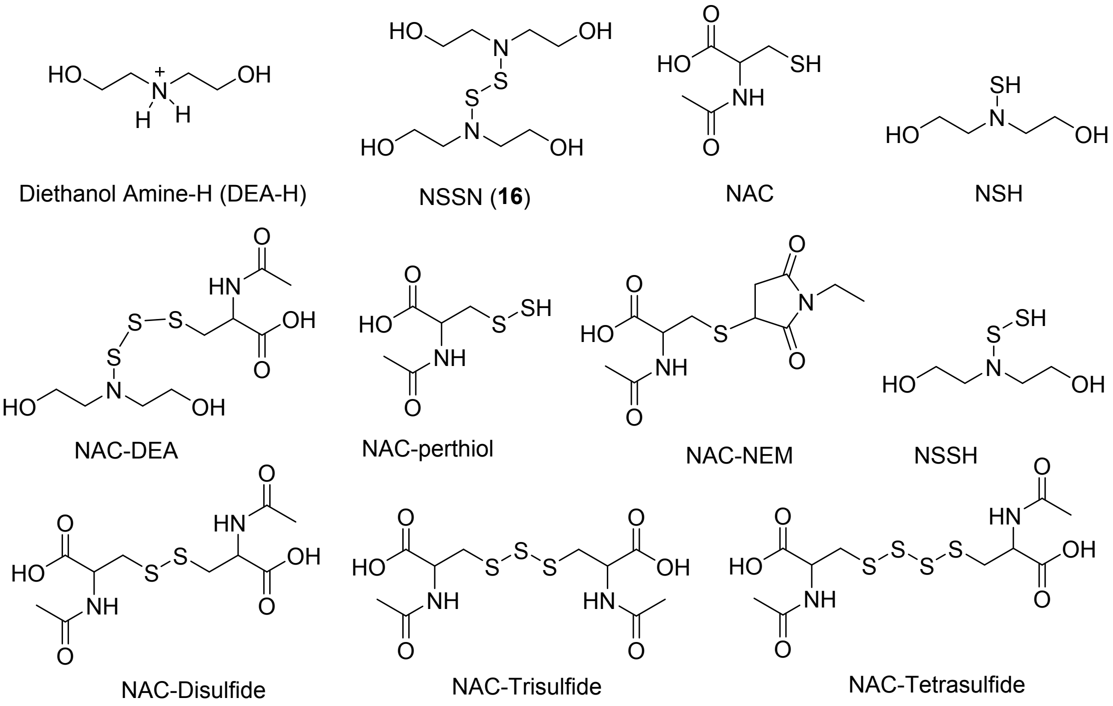


**Figure S5.** Observed and predicted compounds in HRMS studies based on predicted intermediates and abbreviations used.

**Table S4.** HRMS calculated and found values for starting materials used in all reactions. Values reported for observed cation pairings ($Na^+$, $K^+$, DEA-H).

| Starting Materials | | Na+ (22.9988) | K+ (38.9637) | 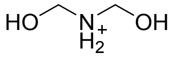 (106.0864) |
|---|---|---|---|---|
| NSSN (**16**) | **Found** | 295.0750 | 311.0492 | - |
| | **Calc.** | 295.0844 | 311.0537 | - |
| NAC | **Found** | 186.0192 | - | - |
| | **Calc.** | 186.0244 | - | - |

**Table S5.** HRMS calculated and found values for observed intermediates for experiment with 2 equiv. NAC. Values reported for observed cation pairings ($Na^+$, $K^+$, DEA-H). Full HRMS data below (**Figure S6-S10**)

| 2x NAC | | Na+ (22.9988) | K+ (38.9637) | 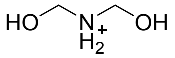 (106.0864) |
|---|---|---|---|---|
| NAC-NEM | **Found** | 311.0672 | 327.0405 | 394.1664 |
| | **Calc.** | 311.0744 | 327.0437 | 394.1664 |
| NAC-DEA-Trisulfide | **Found** | 353.0272 | 369.0003 | 436.1234 |
| | **Calc.** | 353.0344 | 369.0037 | 436.1264 |

**Table S6.** HRMS calculated and found values for observed intermediates for experiment with 5 equiv. NAC. Values reported for observed cation pairings ($Na^+$, $K^+$, DEA-H). Full HRMS data below (**Figure S11-S15**)

| 5x NAC | | Na+ (22.9988) | K+ (38.9637) | 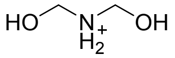 (106.0864) |
|---|---|---|---|---|
| NAC-NEM | **Found** | 311.0665 | 327.0405 | 394.1664 |
| | **Calc.** | 311.0744 | 327.0437 | 394.1664 |
| NAC-Disulfide | **Found** | 347.0331 | - | 430.1301 |
| | **Calc.** | 347.0344 | - | 430.1264 |
| NAC-DEA-Trisulfide | **Found** | 353.0260 | - | 436.1229 |
| | **Calc.** | 353.0344 | 369.0037 | 436.1264 |
| NAC-Trisulfide | **Found** | 379.0051 | - | 462.1020 |
| | **Calc.** | 379.0144 | - | 462.1064 |
| NAC-Tetrasulfide | **Found** | - | - | 494.0742 |
| | **Calc.** | - | - | 494.0764 |

**Table S7.** HRMS calculated and found values for observed intermediates for experiment with 10 equiv. NAC. Values reported for observed cation pairings ($Na^+$, $K^+$, DEA-H). Full HRMS data below (**Figure S16-S20**)

| 10x NAC | | Na+ (22.9988) | K+ (38.9637) | 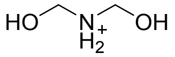 (106.0864) |
|---|---|---|---|---|
| NAC-NEM | **Found** | 311.0663 | 327.0405 | 394.1632 |
| | **Calc.** | 311.0744 | 327.0437 | 394.1664 |
| NAC-Disulfide | **Found** | 347.0332 | 363.0071 | 430.1300 |
| | **Calc.** | 347.0344 | 363.0037 | 430.1264 |
| NAC-DEA-Trisulfide | **Found** | 353.0263 | - | 436.1229 |
| | **Calc.** | 353.0344 | - | 436.1264 |
| NAC-Trisulfide | **Found** | 379.0052 | 394.1632 | 462.1020 |
| | **Calc.** | 379.0144 | - | 462.1064 |
| NAC-Tetrasulfide | **Found** | - | - | 494.0742 |
| | **Calc.** | - | - | 494.0764 |

**Table S8.** Observed intermediates in HRMS assignments for individual HRMS data below. Categorized intermediate structure (color) and cation pairing (shape) $Na^+$ (circle), $K^+$ (square) , DEA-H (triangle).

| | Na+ | K+ | DEA-H |
|---|---|---|---|
| 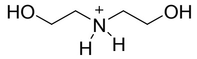 ● | - | - | - |
| 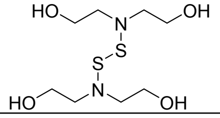 | ○ | □ | △ |
| 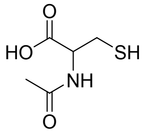 | ○ | □ | △ |
| 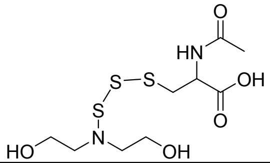 | ○ | □ | △ |
| 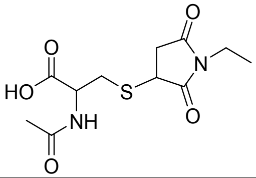 | ○ | □ | △ |
| 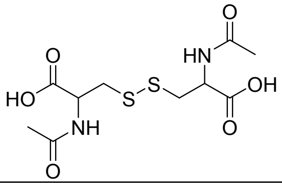 | ○ | □ | △ |
| 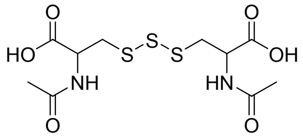 | ○ | □ | △ |
| 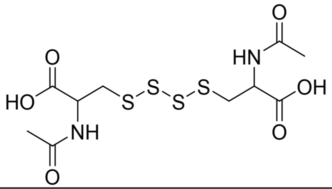 | ○ | □ | △ |

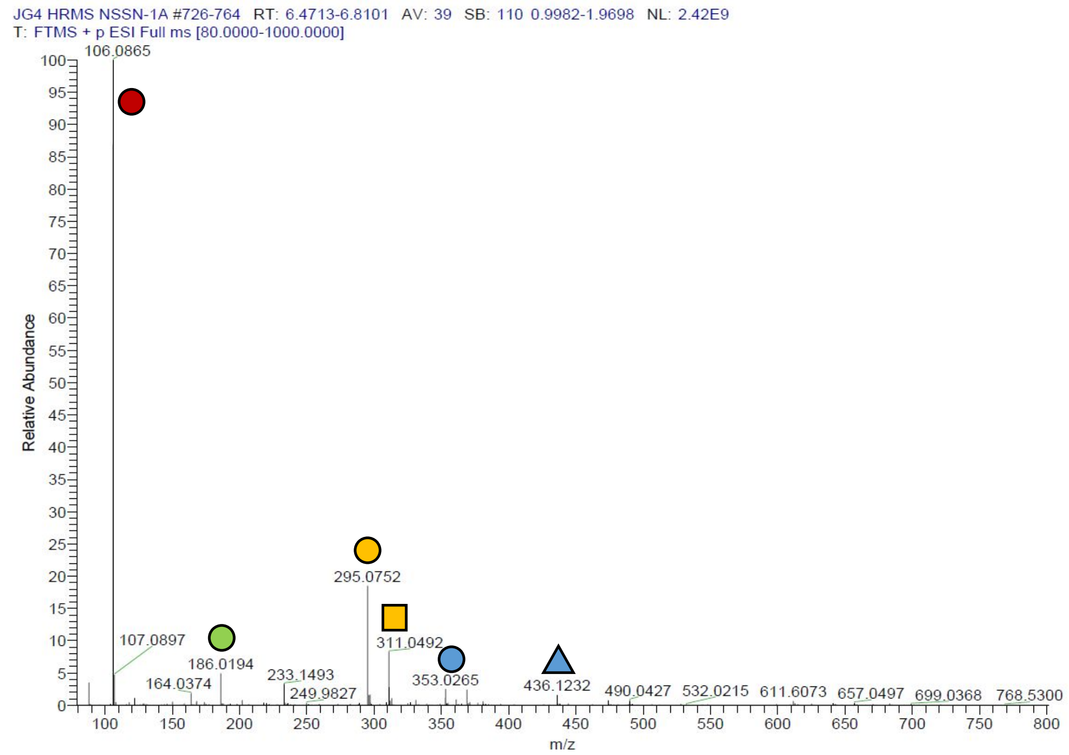


**Figure S6.** ESI HRMS of NSSN (0.1 mM) with NAC (2 equiv.) after 5 min.

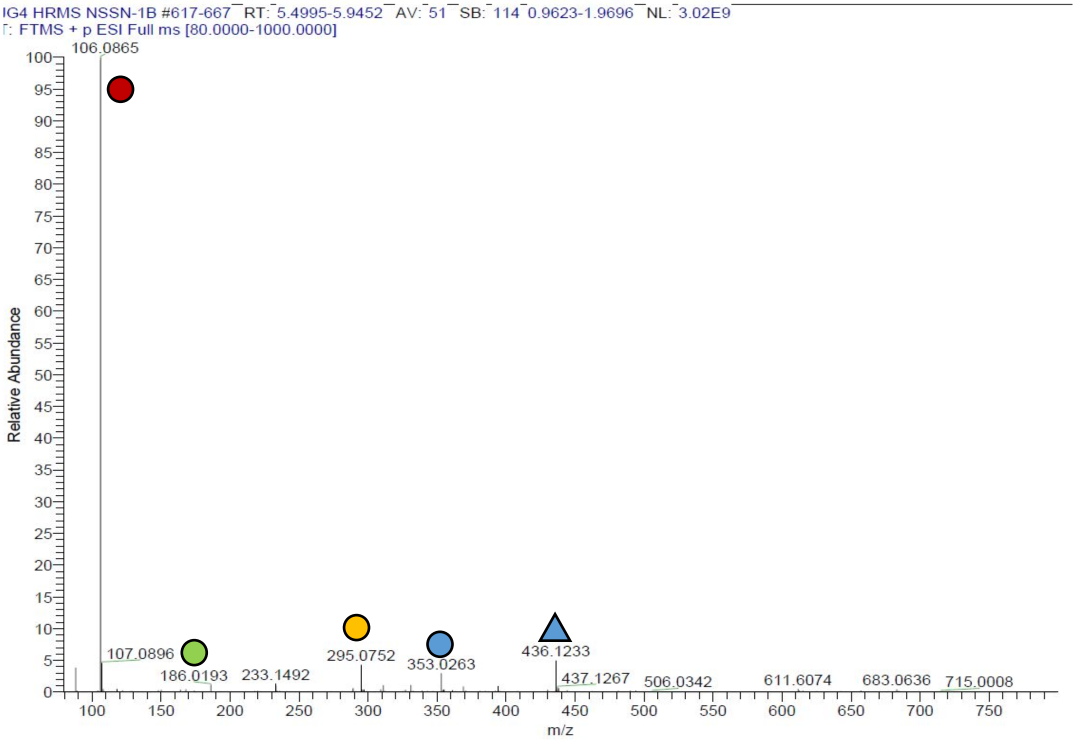


**Figure S7.** ESI HRMS of NSSN (0.1 mM) with NAC (2 equiv.) after 20 min.

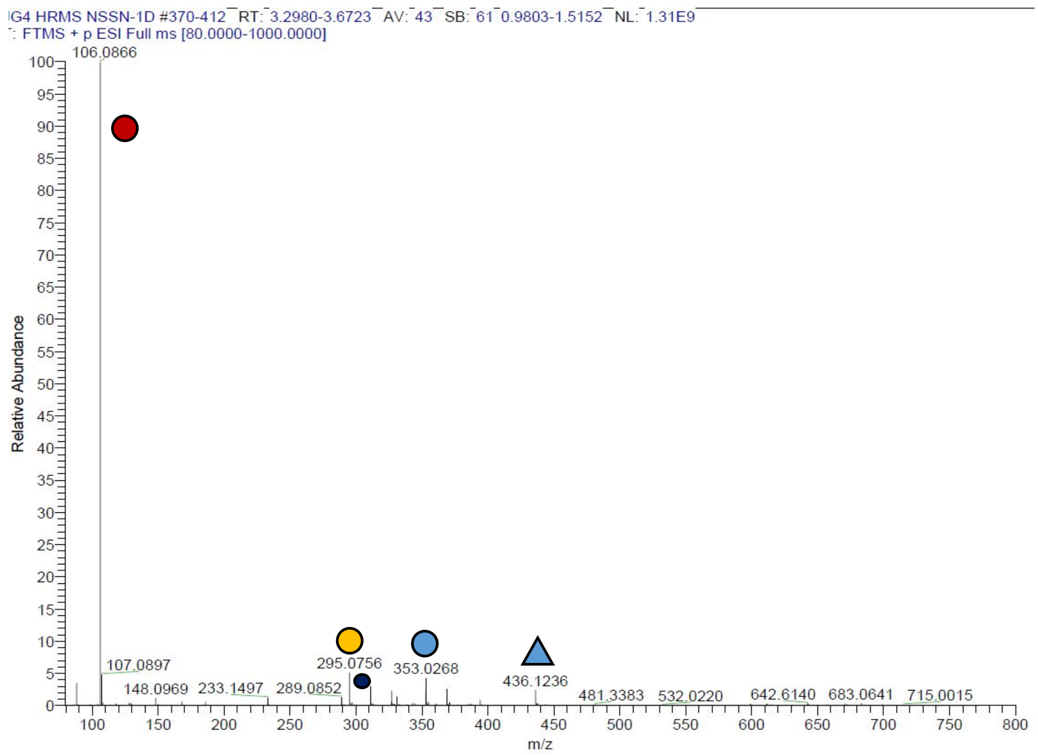


**Figure S8.** ESI HRMS of NSSN (0.1 mM) with NAC (2 equiv.) after 75 min.

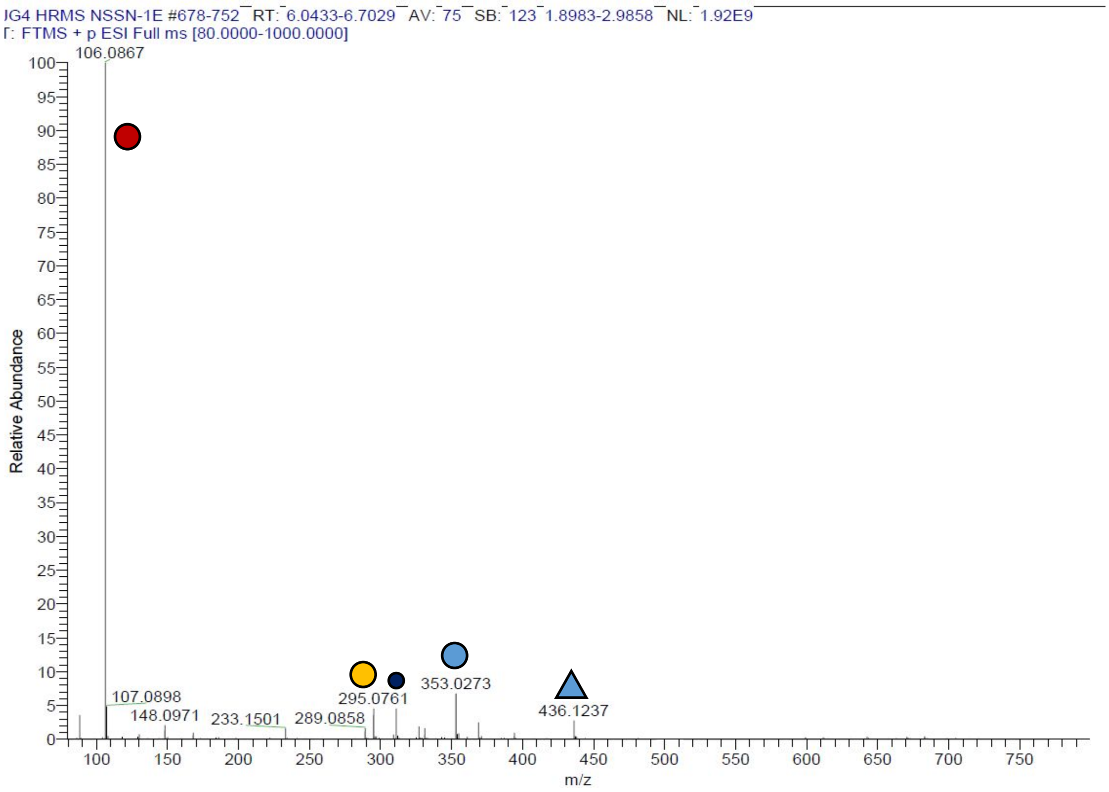


**Figure S9.** ESI HRMS of NSSN (0.1 mM) with NAC (2 equiv.) after 2.5 h.

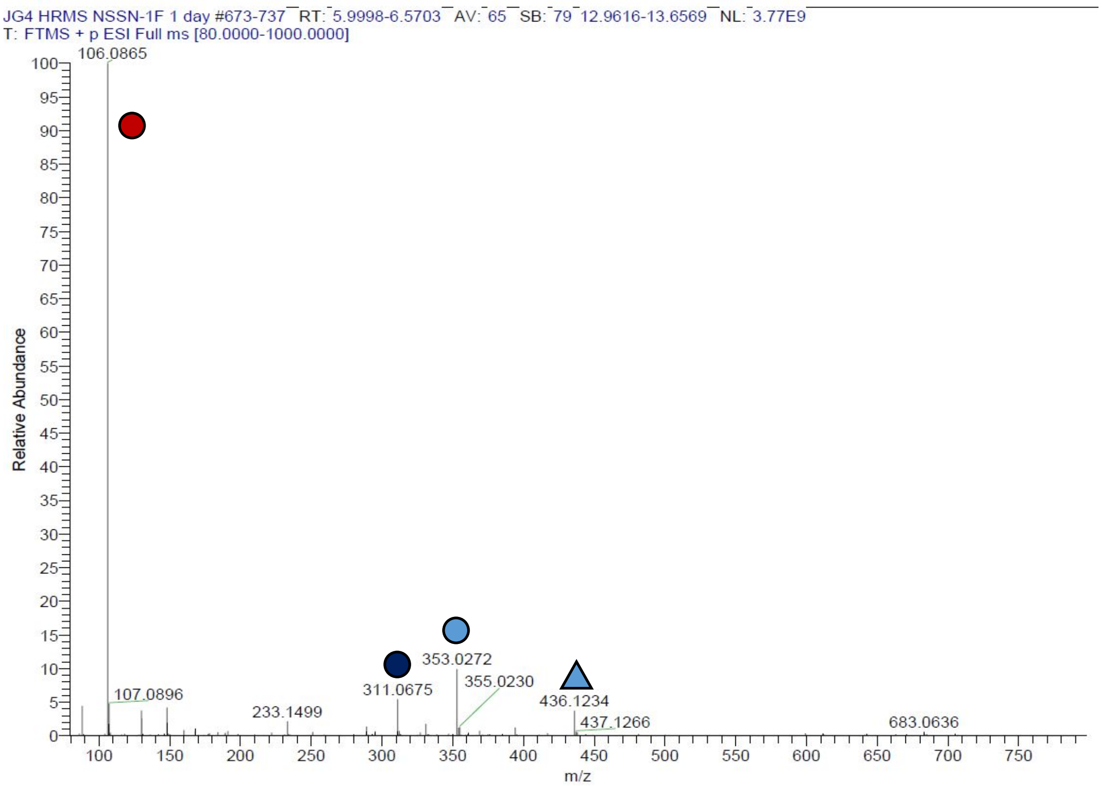


**Figure S10.** ESI HRMS of NSSN (0.1 mM) with NAC (2 equiv.) after 24 h.

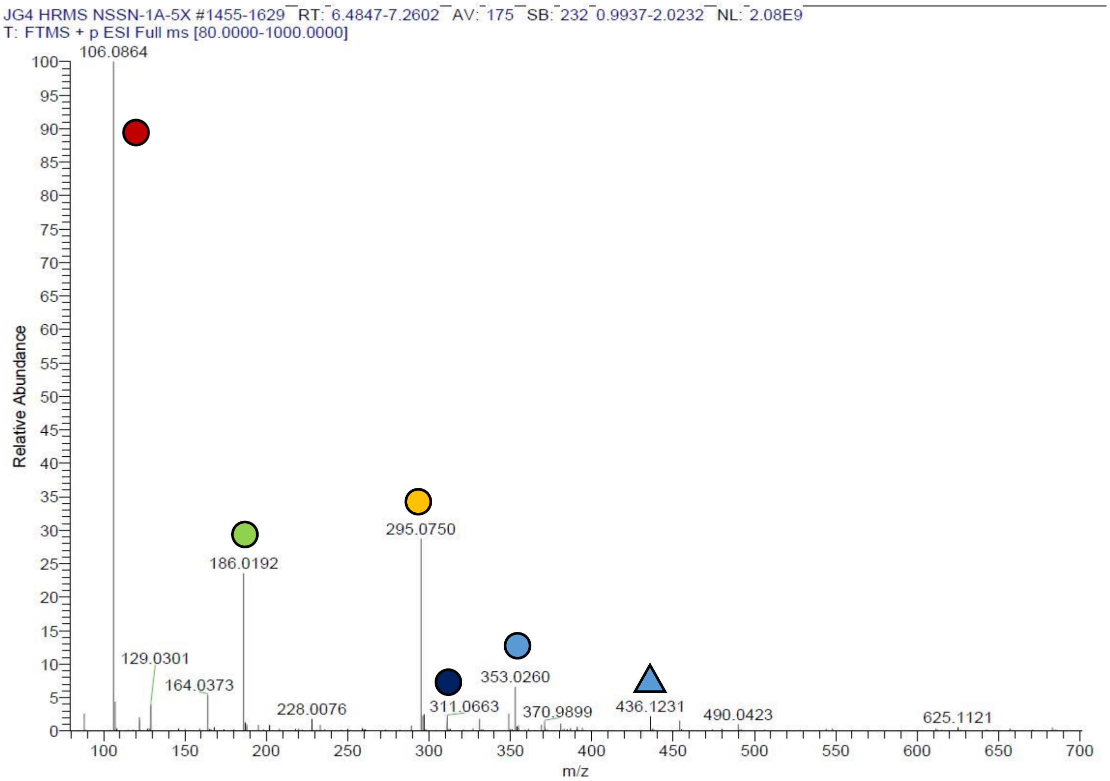


**Figure S11.** ESI HRMS of NSSN (0.1 mM) with NAC (5 equiv.) after 5 min.

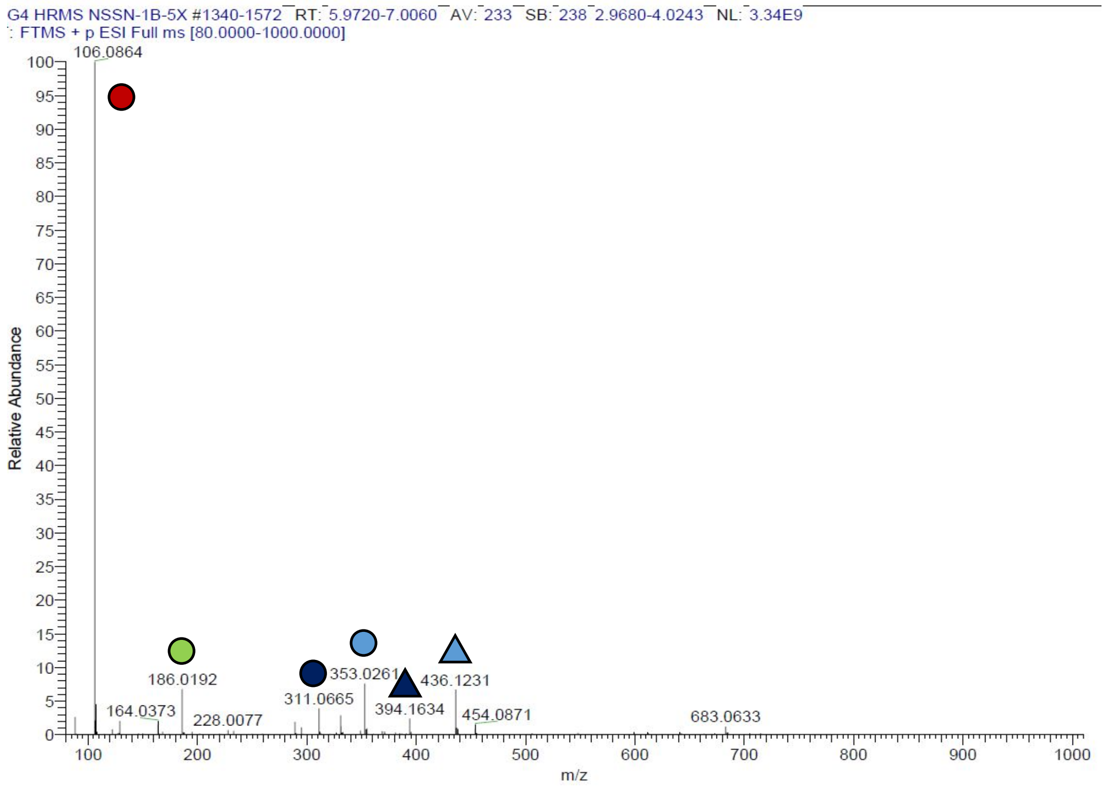


**Figure S12.** ESI HRMS of NSSN (0.1 mM) with NAC (5 equiv.) after 20 min.

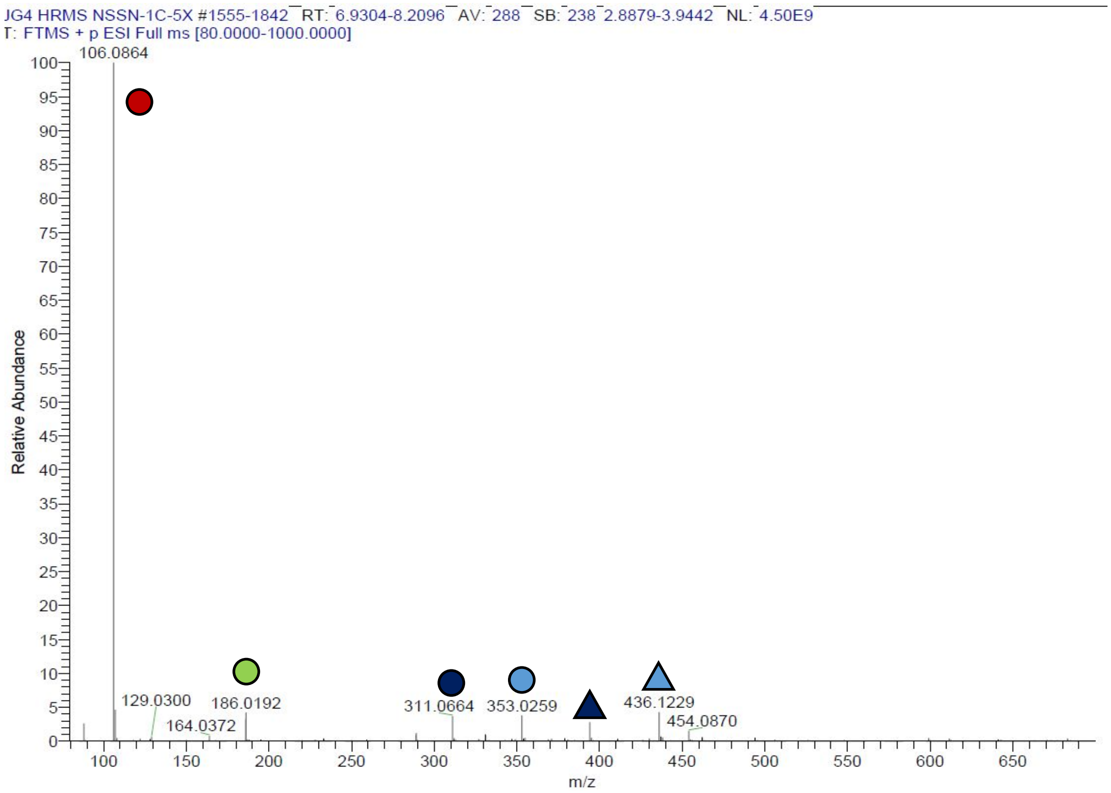


**Figure S13.** ESI HRMS of NSSN (0.1 mM) with NAC (5 equiv.) after 75 min.

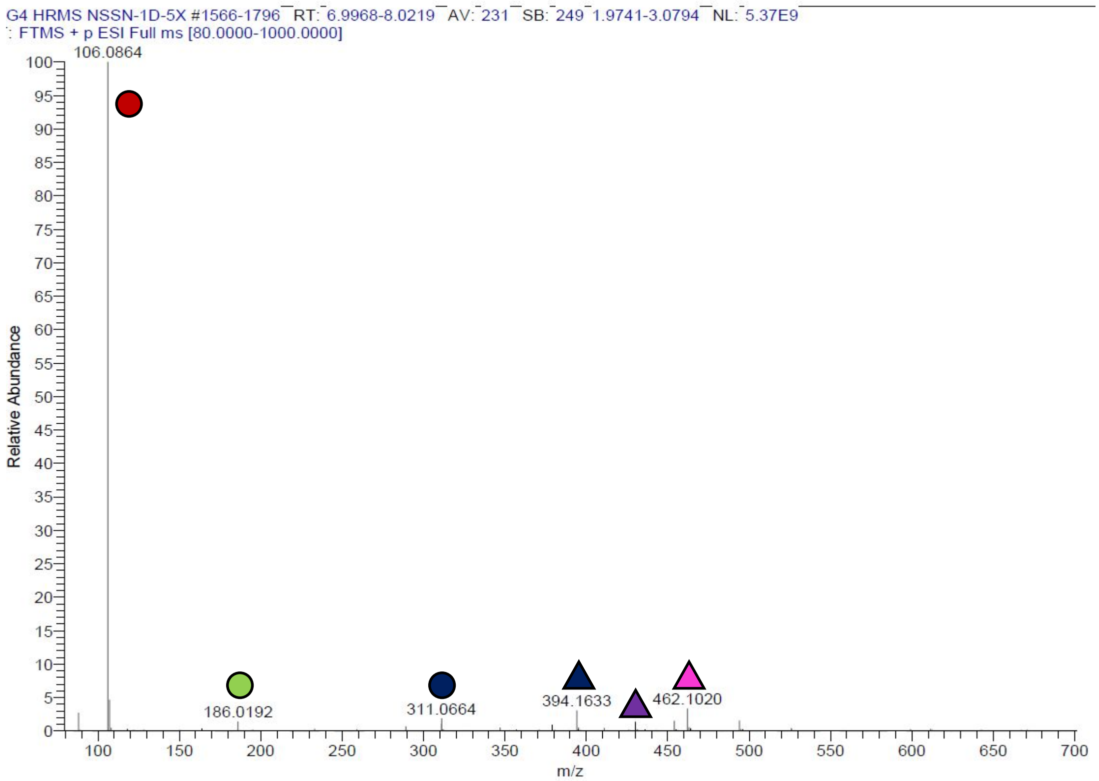


**Figure S14.** ESI HRMS of NSSN (0.1 mM) with NAC (5 equiv.) after 2.5 h.

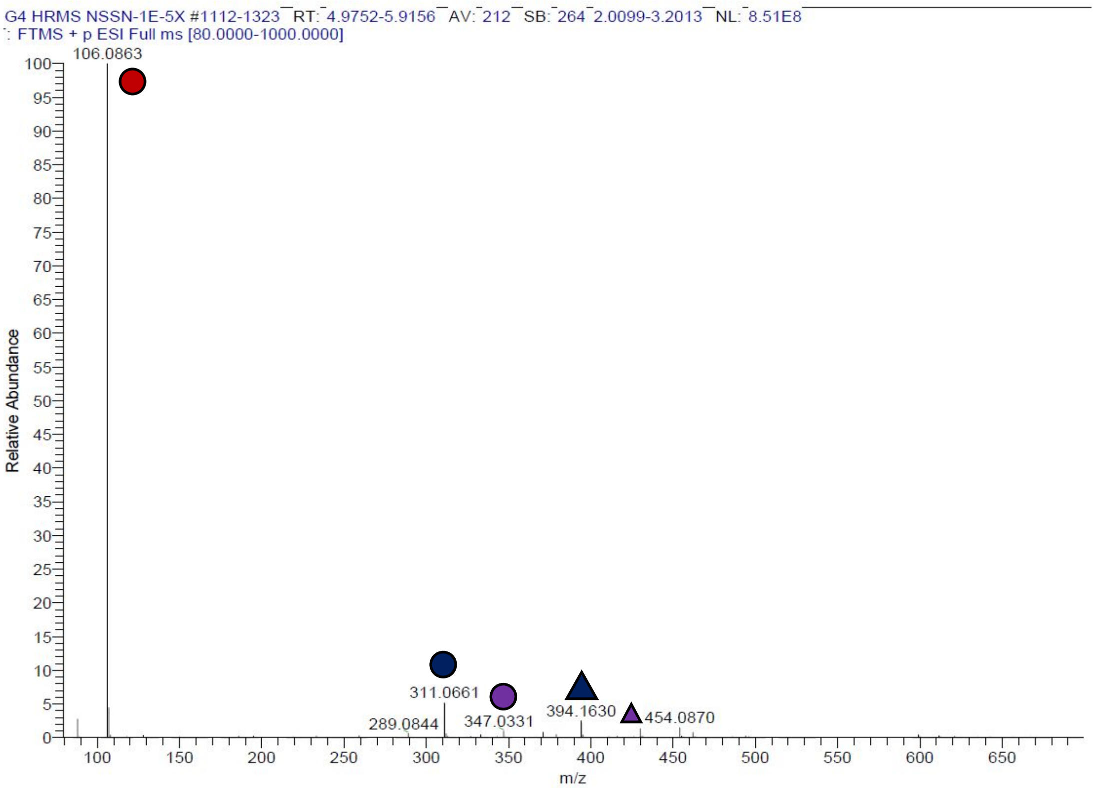


**Figure S15.** ESI HRMS of NSSN (0.1 mM) with NAC (5 equiv.) after 24 h.

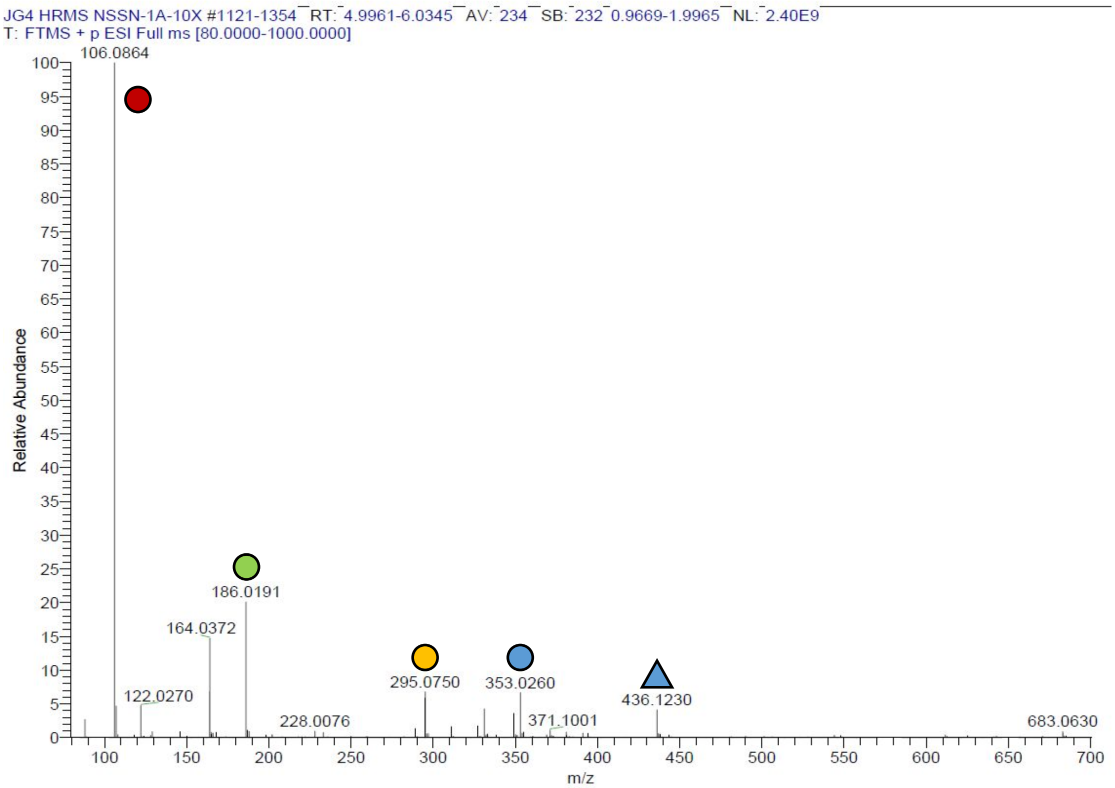


**Figure S16.** ESI HRMS of NSSN (0.1 mM) with NAC (10 equiv.) after 5 min.

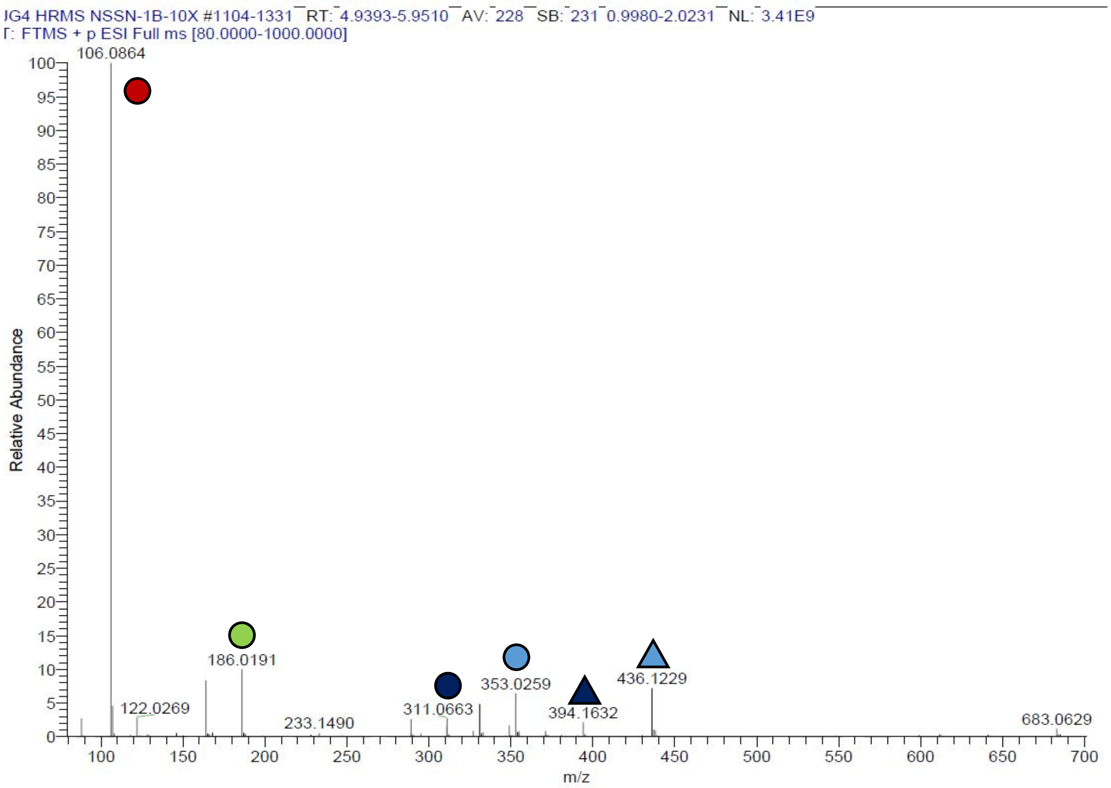


**Figure S17.** ESI HRMS of NSSN (0.1 mM) with NAC (10 equiv.) after 20 min.

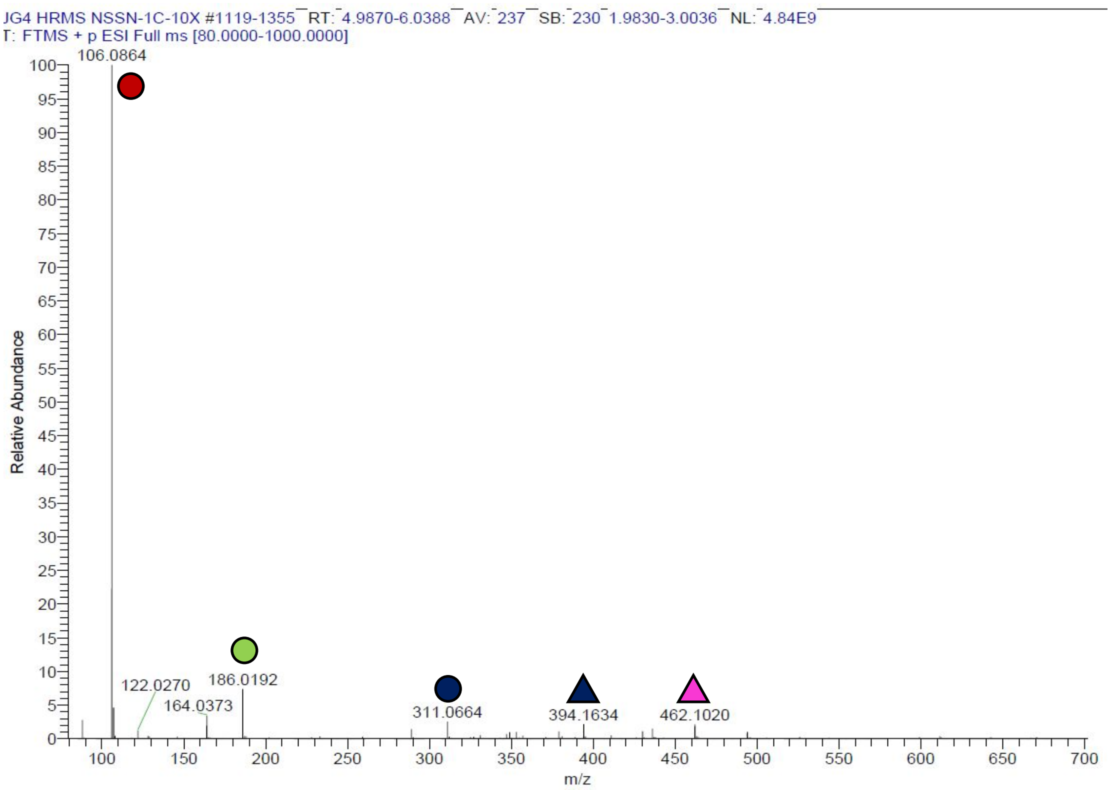


**Figure S18:** ESI HRMS of NSSN (0.1 mM) with NAC (10 equiv.) after 75 min.

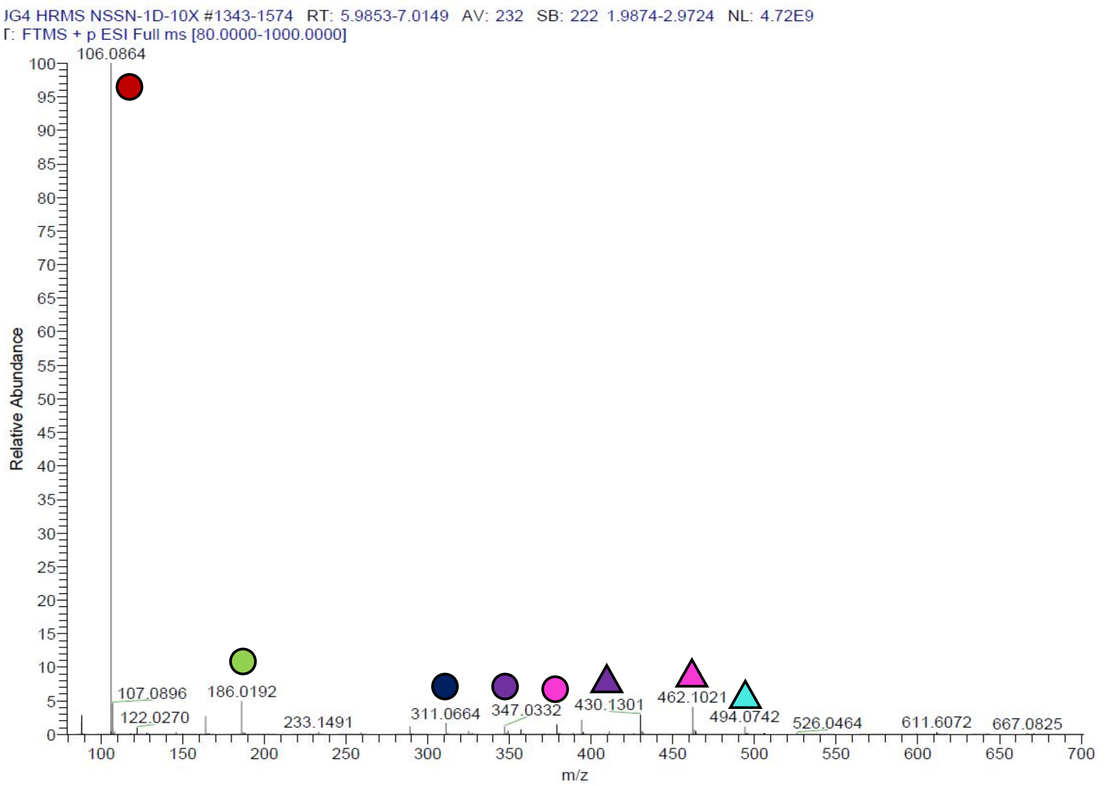


**Figure S19.** ESI HRMS of NSSN (0.1 mM) with NAC (10 equiv.) after 2.5 h.

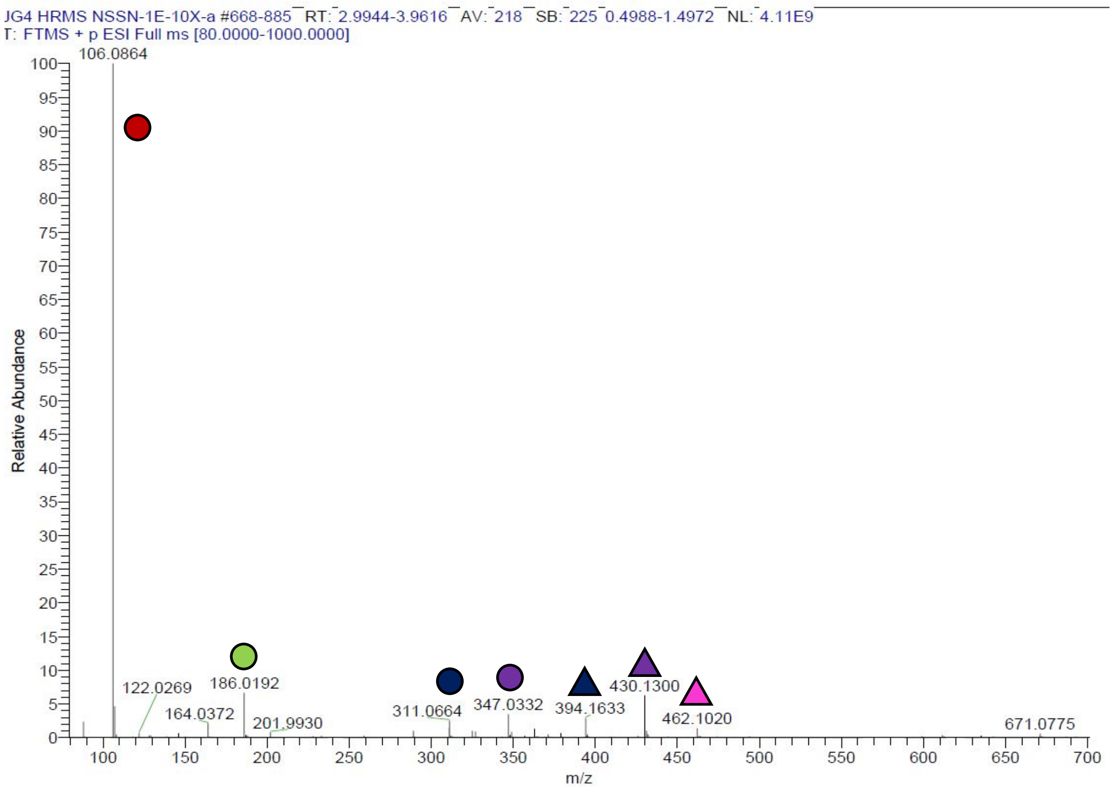


**Figure S20.** ESI HRMS of NSSN (0.1 mM) with NAC (10 equiv.) after 24 h.

**Spectroscopic Data:**

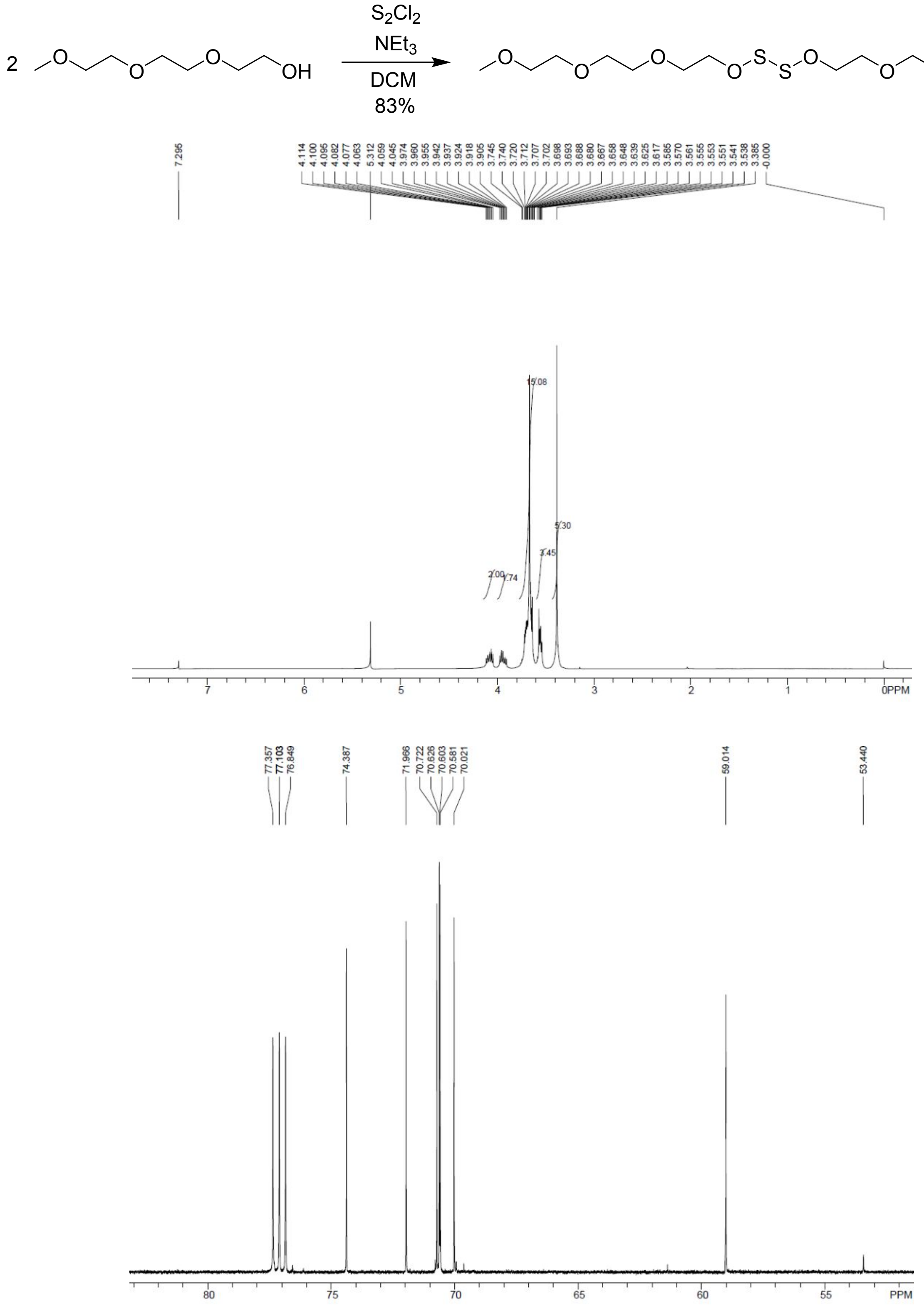


**Figure S21.** Synthetic scheme, $^1$H NMR spectrum and $^{13}$C NMR spectrum of **12.**

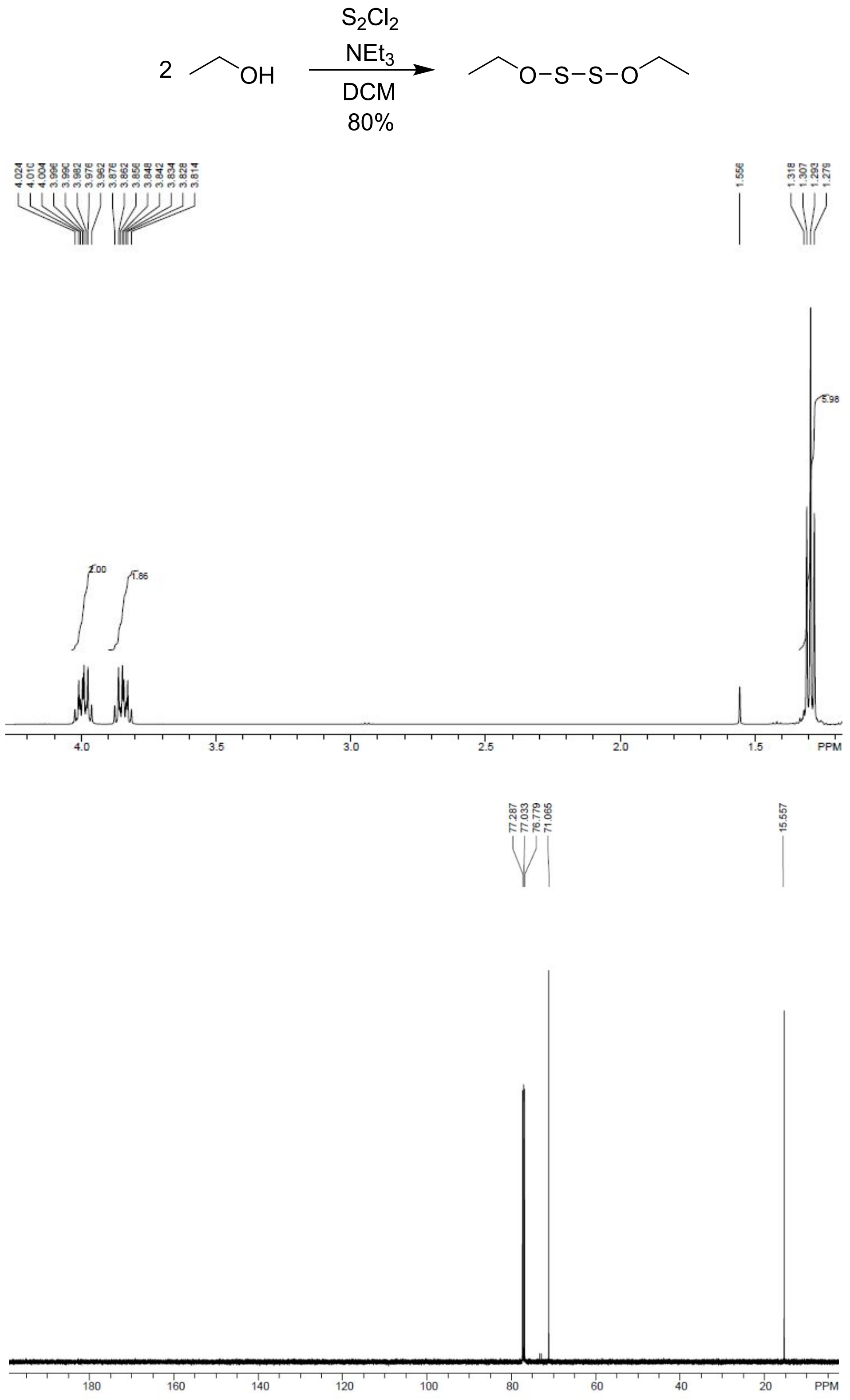


**Figure S22.** Synthetic scheme, $^{1}H$ NMR spectrum and $^{13}C$ NMR spectrum of **10.**

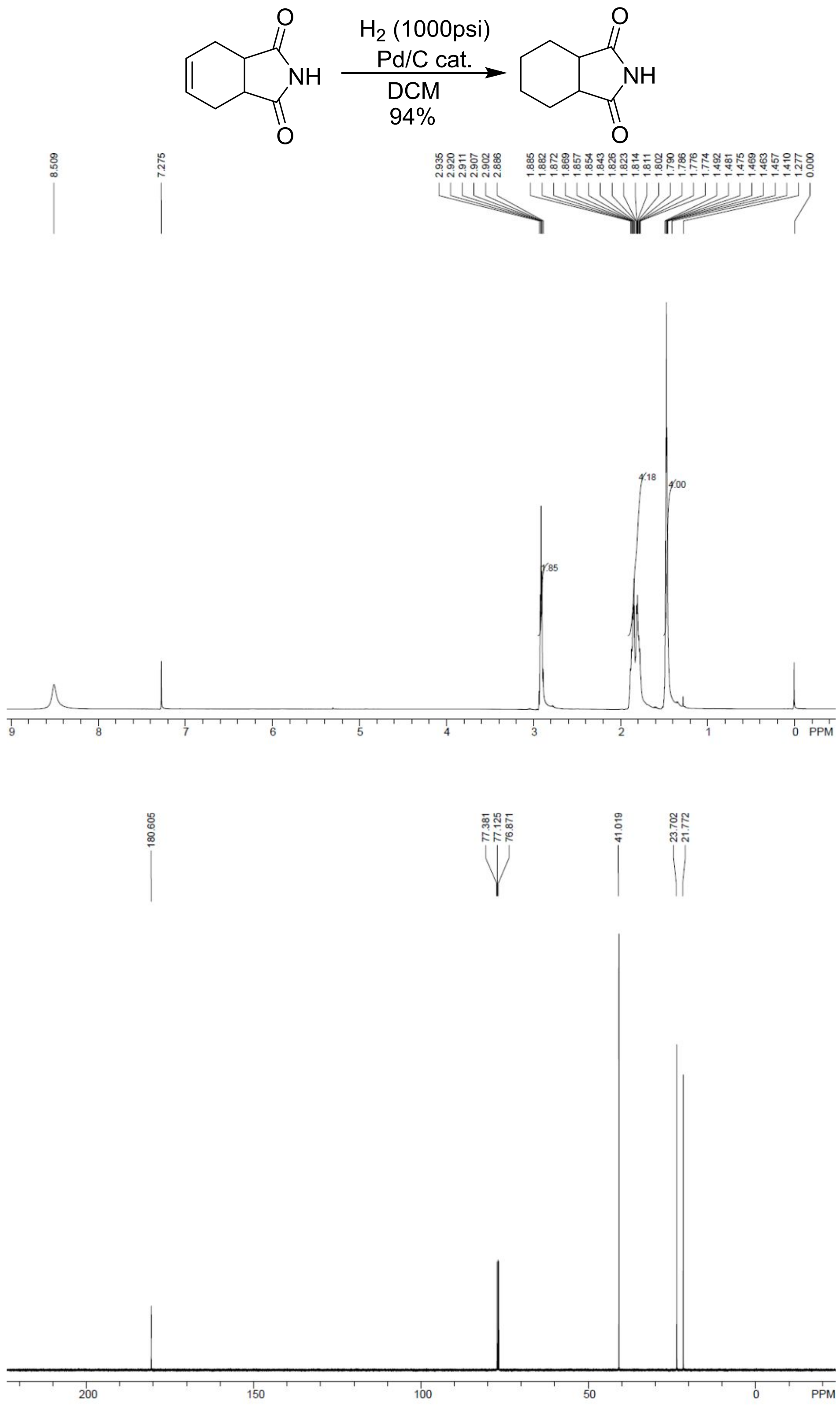


**Figure S23.** Synthetic scheme, $^{1}H$ NMR spectrum and $^{13}C$ NMR spectrum of cis-1,2,3,4,5,6-Hexahydrophthalimide.

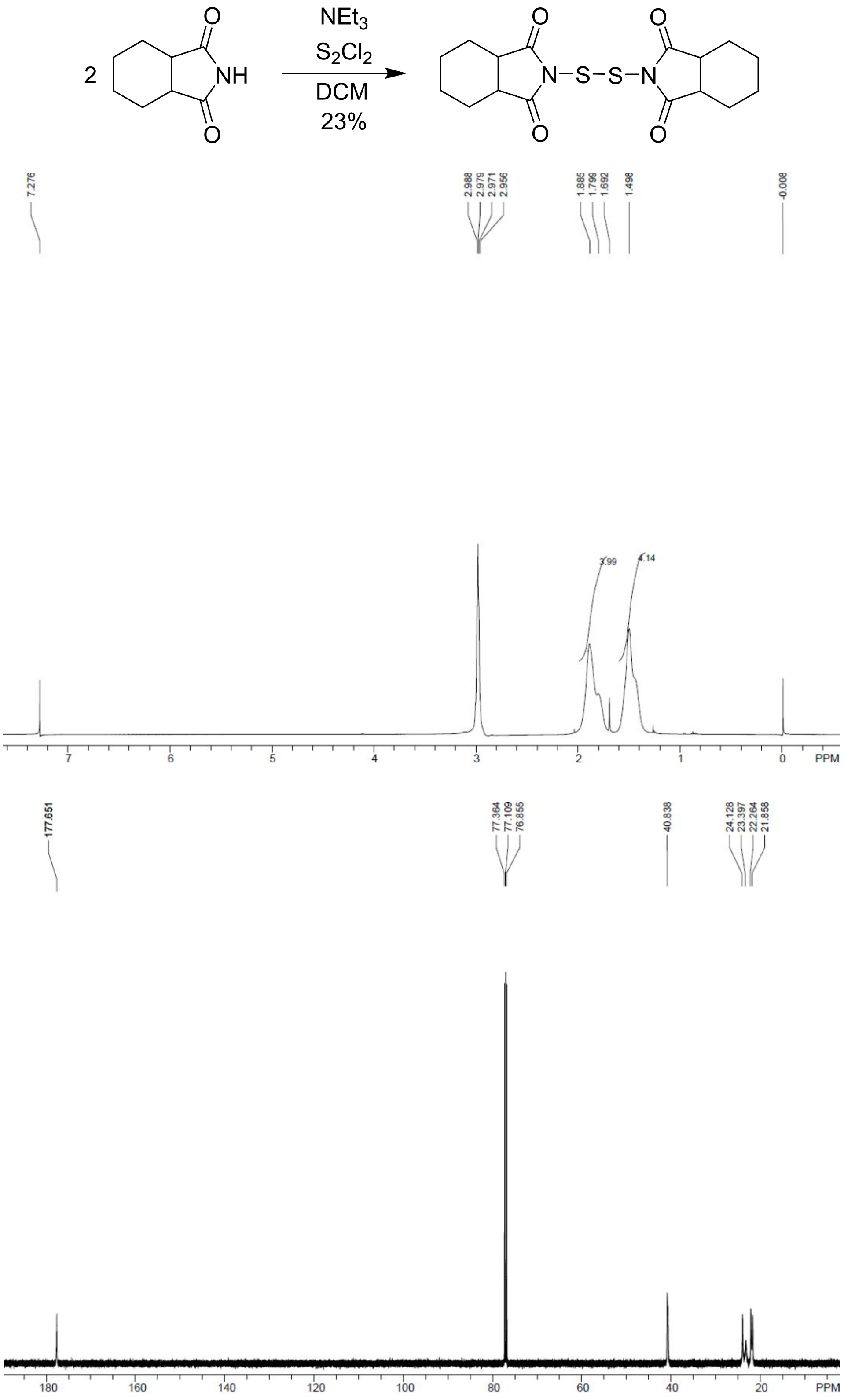


**Figure S24.** Synthetic scheme, $^{1}H$ NMR spectrum and $^{13}C$ NMR spectrum of **15.**

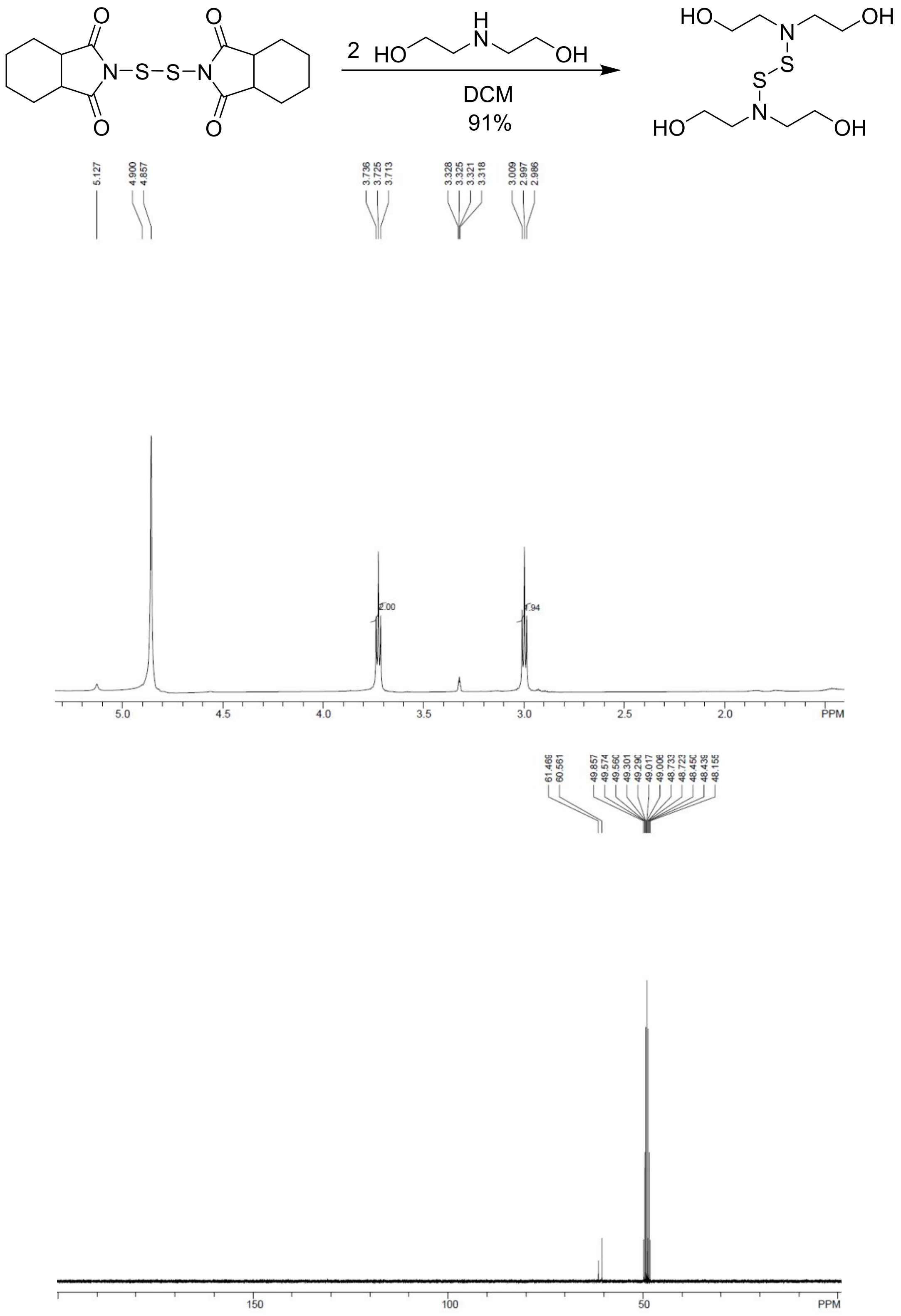


**Figure S25.** Synthetic scheme, $^{1}H$ NMR spectrum and $^{13}C$ NMR spectrum of **16.**

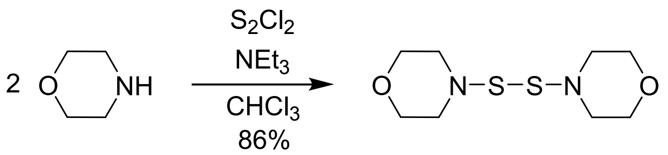


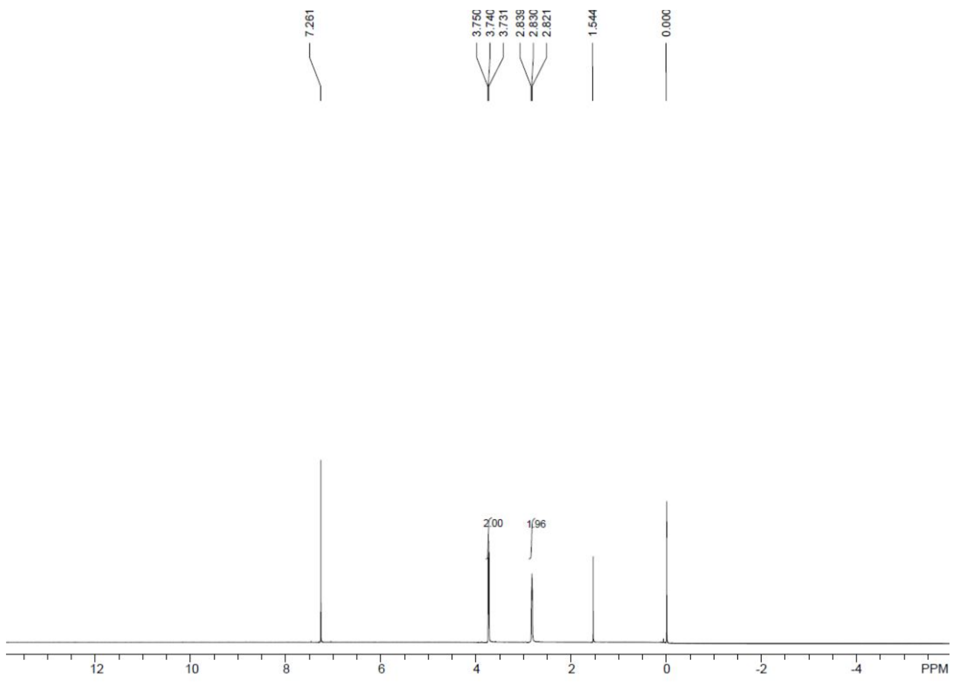

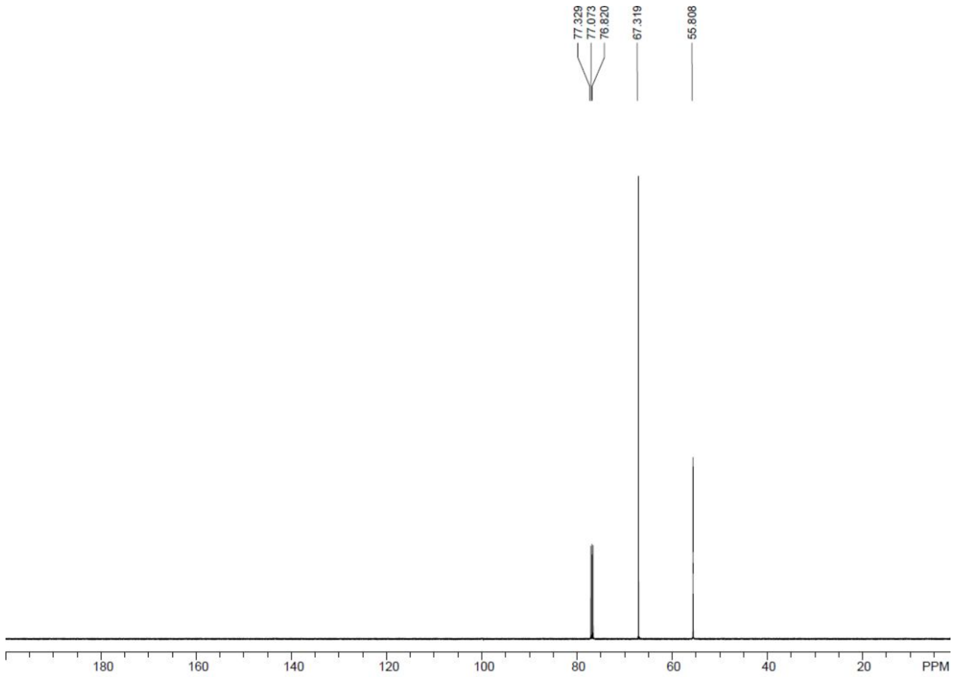

**Figure S26.** Synthetic scheme, $^{1}H$ NMR spectrum and $^{13}C$ NMR spectrum of **14.**

1. Tyler A. Graf, Jun Yoo, Adam B. Brummett, Ran Lin, Markus Wohlgenannt, Daniel Quinn, and Ned B. Bowden *Macromolecules* **2012** *45* (20), 8193-8200.

2. Barton, D. H. R.; Ley, S. V.; Magnus, P. D., Stability of N,N-dialkylthiohydroxylamines. *J. Chem. Soc.*, Chem. Commun. 1975, (20), 855-856.
